# Patients’ and Physicians’ Perceptions of AI Integration in Prostate Cancer Diagnosis: Mixed Methods Study of Challenges to the Patient-Physician Relationship

**DOI:** 10.2196/101751

**Published:** 2026-09-30

**Authors:** Ekaterina Koshmanova, Delphine Kirkove, Bernard Voz, Nicolas Gillain, Aurélie Matagne, Louise Bruwier, David Waltregny, Benoît Pétré

**Affiliations:** 1 Department of Information System Management, University Hospital of Liège, CHU de Liège, Avenue de l'Hôpital, 1, Liège, 4000, Belgium, +32 4 323 33 50; 2 Department of Public Health Sciences, Faculty of Medicine, University of Liège, CHU de Liège, Liège, Belgium; 3 Department of Urology, University Hospital of Liège, CHU de Liège, Liège, Belgium

**Keywords:** prostate cancer, diagnostic imaging, trust, uncertainty, clinical decision-making, patient-physician relationship, mixed methods, health care professionals, patients' and physicians' perspectives

## Abstract

**Background:**

AI is increasingly integrated into prostate cancer diagnostics, with the potential to improve accuracy and efficiency. However, it also raises important questions about the conditions and barriers that may influence its successful implementation in this clinical context.

**Objective:**

This study aimed to examine how patients and physicians perceived the integration of AI in prostate cancer diagnostics, with particular attention to its impact on the clinical relationships and the roles of patients and physicians.

**Methods:**

A sequential explanatory mixed methods design was used. Quantitative data were collected using an online questionnaire administered to patients with localized prostate cancer (N=51). Descriptive analyses focused on perceived benefits, willingness to use AI, and associated concerns. Qualitative data were collected through focus groups and semistructured interviews with patients (n=16) and physicians (n=11). Data were analyzed using an iterative, inductive thematic analysis.

**Results:**

Quantitative findings showed that despite recognizing the potential benefits of AI, patients remained divided regarding the use of such tools in their own care. Qualitative findings suggested that this hesitation cannot be explained solely in terms of perceived performance or utility. Rather than simply reducing complexity in clinical decision-making, AI appeared to reconfigure the certainties on which trust within the patient-physician relationship was established. This reconfiguration was reflected across epistemic, ethical, and role-related dimensions. Patients emphasized difficulties in understanding AI-generated knowledge, whereas physicians focused on issues of reliability, validation, and clinical relevance. Ethical concerns centered on responsibility, which was consistently attributed to physicians, while errors made by AI were perceived as less acceptable than those made by physicians. Role-related uncertainties were reflected in ambivalent patient positions: while some participants sought more information to remain involved in decision-making, others preferred to rely on physicians, reflecting variation in how patients engaged with complex clinical information. AI was generally viewed as a supportive tool rather than a replacement for clinical judgment, while its integration was associated with evolving professional roles, including increased demands for interpretation, communication, and oversight.

**Conclusions:**

The integration of AI in prostate cancer diagnostics is shaped not only by its technical performance but also by how it interacts with trust within the patient-physician relationship. Our findings suggest that AI may reshape, rather than eliminate, uncertainty related to knowledge, responsibility, and social roles. Its integration into clinical practice therefore requires careful attention to clinician oversight, communication, and the relational context in which decisions are made.

## Introduction

AI is increasingly integrated into clinical medicine to support diagnostic and decision-making processes across diverse health care settings [1] and has the potential to enhance diagnostic accuracy, standardize interpretation of complex clinical data, and optimize patient care, while also reshaping workflows in health care systems [2].

Yet, the translation of AI systems from research environments into routine clinical practice remains challenging. Beyond technical performance, AI adoption is constrained by practical and ethical barriers, including data privacy, algorithmic bias, limited interpretability, workflow integration challenges, and concerns regarding accountability and patient consent [3].

However, even when these technical and regulatory challenges are addressed, successful implementation of AI in clinical practice depends on a broader set of social and organizational dynamics, including how different stakeholders understand, interpret, and engage with these technologies. In general health care contexts, patients express positive attitudes toward AI, tempered by concerns about data privacy, transparency, accountability, and the preservation of human-centered care [4-7]. Among health care professionals, the acceptance and use of AI are shaped by task type, perceived clinical value, and anticipated impact on professional roles and responsibilities [8,9] and may be limited by distrust in AI and unclear regulatory standards [10]. Asan et al [11] identified key factors shaping trust in AI as a central psychological mechanism influencing clinicians’ willingness to use it, such as perceived reliability, interpretability, and prior experience, which are critical for successful AI integration into clinical workflows. Patients’ trust, in turn, depends more on the physician-patient relationship and increases when AI operates under clear physician supervision [12,13]. Patients are therefore willing to consider AI as an assistive tool if human interaction is preserved, data transparency is ensured, and privacy is protected [14]. Extending these findings, an integrative review of hospital-based AI implementation emphasizes the importance of organizational readiness, training, infrastructure, and concerns regarding professional autonomy as critical determinants of sustainable adoption [15].

Given the increasing application of AI in prostate cancer diagnostics, it is essential to examine how these tools are understood and incorporated into clinical practice by both patients and health care professionals, who are the ultimate end users, as well as the conditions and barriers that may influence successful implementation in this specific clinical context. In prostate cancer, AI-assisted imaging and predictive models can enhance imaging analysis and cancer detection [16,17], improve the detection of clinically significant tumors, and optimize biopsy decisions [18]. Such approaches aim to enhance risk stratification while reducing unnecessary invasive procedures such as biopsy, thereby improving the overall efficiency of the diagnostic pathway [19].

Recent studies examining patient perspectives on AI in prostate cancer care indicate that trust and acceptance are higher when AI functions as a supportive tool alongside clinicians rather than autonomously [20,21]. Focus group research further shows that men’s expectations of AI combine hopes for improved diagnostic accuracy with concerns about clinician-patient communication, with acceptance varying depending on whether AI is perceived as a tool, an advanced machine, or a replacement for the physician [22]. Additionally, it has been shown that patients with prostate cancer who had a higher level of education and a greater understanding of AI’s potential were more likely to engage with AI-based interventions, emphasizing the importance of patient education and communication to foster acceptance [23].

Although a growing body of research has examined the acceptability of AI in health care, both in general and in the context of prostate cancer, important gaps remain. First, most studies have focused on either patients or health care professionals separately, thereby overlooking how these perspectives interact within real-world clinical settings. Second, existing research often relied on hypothetical scenarios or general attitudes toward AI, rather than examining how these technologies are understood and engaged with in concrete diagnostic pathways. Third, while trust has been identified as a central factor, less attention has been paid to how AI reshapes clinical relationships, particularly in terms of role distribution, responsibility, and shared decision-making. These limitations are particularly relevant in prostate cancer diagnostics, where uncertainty is inherent and where clinical decisions involve complex trade-offs between risks and benefits [24]. In such contexts, the integration of AI cannot be reduced to questions of technical performance or general acceptance but must be understood in relation to the social, relational, and organizational conditions in which it is embedded.

In light of these considerations, this study aimed to examine how patients and physicians engage with the integration of AI in prostate cancer diagnostics. Specifically, it investigated their willingness to adopt these technologies and explored how such integration was perceived to affect clinical relationships and the roles of both patients and physicians. Combining quantitative and qualitative approaches, the study sought not only to assess levels of acceptance but also to provide an in-depth understanding of the social and relational dynamics shaping the use of AI in this context.

To achieve this, our study was guided by two specific research questions: (1) to what extent are patients with localized prostate cancer willing to accept the use of AI-based diagnostic tools in their care, and how do physicians perceive the conditions influencing the adoption of such tools in clinical practice? and (2) how is the integration of AI perceived to reconfigure the patient-physician relationship and reshape the clinical and professional roles within this relationship?

## Methods

### Overview

This study is part of the FLUTE (Federated Learning and Multi-Party Computation Techniques for Prostate Cancer) project, a Horizon Europe initiative developing trustworthy AI for prostate cancer diagnostics using federated learning to enable multicenter model training while preserving data privacy. The study was registered on ClinicalTrials.gov (NCT07074405). Although the registered study was originally designed as a multinational study, this analysis was restricted to data collected in Belgium at CHU de Liège. This paper reports on the registered objectives concerning patients’ and physicians’ acceptability of AI-supported prostate cancer diagnosis. The registered objective concerning patients’ willingness to share health data for AI development was not addressed in this analysis.

This study used a mixed methods design with a sequential explanatory approach [25]. Quantitative data were collected through a self-administered online questionnaire among patients, while qualitative data were obtained through semistructured interviews and focus groups with patients and physicians. Quantitative findings informed the qualitative phase, as the survey results were used to refine and adapt the interview guides. Quantitative and qualitative findings were integrated at the interpretation stage to provide a comprehensive understanding of attitudes toward AI in prostate cancer diagnosis and care and its impact on the patient-physician relationship, in line with recent approaches for appraising the quality of integration in mixed methods research [26]. To ensure a rigorous and explicit connection between the 2 phases, a comprehensive joint display was constructed at the final interpretive stage. This joint display mapped the initial quantitative benchmarks and theoretical frameworks directly to the verbatim qualitative protocol items and the dimensions of uncertainty, concluding with integrated meta-inferences that synthesized the findings of both methods (Table 1).

**Table 1.** Joint display of sequential explanatory integration: mapping questionnaire benchmarks to interview guides and integrated meta-inferences.

Quantitative findings (n=51)	Connection: informing the qualitative phase	Qualitative themes and empirical evidence (n=27)	Mixed methods integration (meta-inferences)
Divided intention for personal AI use: 45.1% of patients were willing to use AI tools, 45.1% remained uncertain, and 9.8% were unwilling, despite the majority agreeing AI can improve diagnosis.	Patient interview guide (theme 2): “What advantages do you think AI could bring to your medical pathway?” “What risks or concerns seem most important to you regarding AI?” Physician interview guide (theme 2): “What is your current experience or practice with AI in prostate cancer diagnosis?” “What concerns or fears do you personally have regarding the use of AI?”	Epistemic uncertainty and contextual limits Patient view: struggle to grasp complex data sourcing (patient 8: “Where does the information come from?. still can’t quite grasp”). Physician view: inability of the software to manage its own boundary limits or acknowledge doubt (radiologist D7: “The software will never say that it doesn’t know”).	The divided willingness to use AI, despite generally positive views of its potential diagnostic benefits, provides an important context for the epistemic uncertainties identified in the qualitative phase. Participants raised questions about how AI-generated information is produced, interpreted, and evaluated, while clinicians also highlighted limitations related to false positives and uncertainty in clinically ambiguous cases. Together, these findings suggest that positive perceptions of AI’s potential benefits can coexist with uncertainty about how its outputs can be understood and applied in clinical practice.
Anxiety over accountability and error—rated as a high concern by 48% of hesitant or unwilling respondents (n=28).	Patient interview guide (theme 2): “In your opinion, who should be responsible in case of an error in your diagnosis or treatment after the use of AI?” “Do you think the responsibility should lie with the doctor, the AI developer, or both?” Physician interview guide (theme 2): “In your opinion, who should be responsible in the event of an AI-related error?” “Do you inform your patients when you use AI-based tools? If yes, how?”	Ethical uncertainty and asymmetric accountability Patient demand: total reliance on an answerable human agent (patient 2: “Responsibility must not be left unclear. There has to be a physician behind it”). Physician tension: bearing ultimate clinical liability for a system they cannot technically modify or audit (radiologist D9).	Quantitative concerns about responsibility and AI-related errors were further elaborated in the qualitative findings. Patients emphasized the importance of maintaining identifiable human responsibility for clinical decisions, while physicians discussed uncertainty about accountability when AI-generated outputs contribute to decisions for which they remain clinically responsible. Together, these findings suggest that the allocation of responsibility is an important condition shaping participants’ attitudes toward the integration of AI into clinical practice.
Fear of losing human connection—rated as a high concern by 52% of hesitant or unwilling respondents (the highest recorded concern across all domains; n=28).	Patient interview guide (theme 3): “How do you think AI could influence communication and trust with your doctor?” “Are you concerned about a loss of the human connection with your doctor? Why?” Physician interview guide (theme 3): In your opinion, how could AI influence communication, trust, and the physician’s role with patients?" "Patients express concerns that AI could lead to a loss of the human relationship in care. In your opinion, are these concerns justified? Why?”	Social role uncertainty and relational expertise Patient view: irreplaceability of human support during cancer care (patient 15: “People, if they don’t have someone behind them to hold their hand. we are going to lose that”). Physician view: increased commercial, administrative, and evaluative workload without any reduction in clinical hours.	The quantitative concern about a potential loss of human connection was reflected and further contextualized in the qualitative findings. Patients emphasized the importance of human interaction, support, and communication in cancer care, while physicians generally positioned AI as a supportive tool rather than a substitute for their clinical and relational roles. The qualitative findings also suggested that AI may introduce additional responsibilities related to interpreting, supervising, and explaining AI-generated information. Together, these findings indicate that concerns about human connection extend beyond the physical presence of a physician to questions about how professional and relational roles may evolve with AI integration.
High institutional trust versus low familiarity and cautious AI trust: 90.2% of patients rated prior health care experience as good/very good; 86.2% trust health care institutions; 68.6% reported no prior use of AI in diagnostics; 23.5% were unsure; 49% reported low/no familiarity with AI; 39.2% rated technical knowledge as poor/very poor.	Patient interview guide (themes 2 and 3): “Have you ever heard about the use of AI in medical diagnosis?” “How do you think AI could influence communication and trust with your doctor?” Physician interview guide (theme 2): “Have you already used artificial intelligence in your clinical practice? If yes, how.?” “What is your current experience or practice with AI in prostate cancer diagnosis?”	Mediated trust, knowledge gaps, and relational foundations Patient dynamic: cautious due to abstraction; trust is given to the human expert, not the system (patient 1: “It should remain a tool that supports the physician, so you would always have a doctor in front of you”). Physician reality: discrepancies between generalized familiarity and narrow technical knowledge; hesitation to introduce AI into clinical reports (radiologist D4).	The combination of high institutional trust, limited familiarity with AI, and cautious attitudes toward its use provides an important context for interpreting the qualitative findings. Participants frequently positioned the physician as the person responsible for explaining, contextualizing, and overseeing AI-generated information, suggesting that existing trust in physicians may play an important role in how clinical AI is approached. Rather than indicating independent trust or distrust in AI itself, the findings suggest that attitudes toward AI were often considered in relation to existing clinical relationships and professional expertise.

### Ethical Considerations

The study was approved by the Comité d’éthique Hospitalo-Facultaire Universitaire de Liège (Hospital Faculty Ethics Committee of the University of Liège and CHU de Liège, Belgium; ref: 2024/345, 2025/368). All participants provided informed consent prior to participation. Unique codes were assigned to enable data withdrawal requests while preserving anonymity. Quality assurance of the quantitative phase procedures included monitoring for completeness, checking for missing data and logical consistencies, and reviewing free-text responses to remove any inadvertent identifiers. An analysis dataset was derived from the raw exports and documented in an analysis log. Only authorized members of the research team had access to raw data. For the qualitative phase, all participants provided additional written consent for recordings. Sessions were audio-recorded, transcribed verbatim, and pseudonymized using participant codes. Transcripts and recordings were securely stored on password-protected institutional servers with access limited to authorized research team members. Any identifying information was removed during transcription.

### Quantitative Phase

#### Participants and Recruitment

The study population consisted of adults (aged ≥18 y) with localized prostate cancer; exclusion criteria included an inability to speak French, metastatic disease at diagnosis, terminal illness, and severe cognitive impairment.

Participants were recruited at CHU de Liège, a university hospital in Belgium, through flyers distributed in clinical settings and direct invitations from health care professionals (physicians and nurses) involved in the care of patients with prostate cancer. In addition, the researcher (EK) approached patients who were hospitalized after radical prostatectomy and assisted, if needed, with questionnaire completion without influencing responses. Eligible patients were also contacted via phone by a urologist (LB). Participation was voluntary, and patients were informed that their decision would not affect their medical care.

#### Data Collection

The questionnaire was implemented in REDCap (Vanderbilt University), a secure web-based platform for research data collection [27,28] that meets regulatory requirements for health research and is supervised by the Clinical Trial Center at CHU de Liège. Participants accessed the questionnaire via a secure link and were first presented with an information and consent section, followed by eligibility screening and completion of the main questionnaire. Within the information section, all participants were presented with the same standardized hypothetical scenario framing AI as a noninvasive decision support tool that combines blood tests, medical history, and magnetic resonance imaging to evaluate whether a prostate biopsy is required. A separate, unlinked REDCap form was used to collect contact details from participants who volunteered for the qualitative phase.

#### Studied Variables

We designed the questions based on 2 established frameworks for health technology implementation: the Theoretical Framework of Acceptability [29] and the NASSS (nonadoption, abandonment, scale-up, spread, and sustainability) framework [30]. We also included specific questions about the prostate cancer clinical pathway developed by our research team. This study specifically focused on the variables tied to AI acceptance and the patient-physician relationship. The questionnaire was developed in French, and the initial items were reviewed and refined by our research team, which included urologists and radiologists. The tool was then pilot tested with a small group of colleagues to check for language clarity and ease of understanding. The complete questionnaire in French can be found in Multimedia Appendix 1. Perceived benefits of AI-based diagnostic tools were measured using a series of items evaluating agreement with statements such as whether AI could improve prostate cancer diagnosis, advance the diagnostic process, and provide accurate diagnoses (questions 3‐5 in Multimedia Appendix 1). Responses were recorded on 7-point Likert-type scales (ranging from “strongly disagree” to “strongly agree”).

Willingness to use AI-based tools for prostate cancer diagnosis was assessed with a single item (yes, no, or uncertain, question 12 in Multimedia Appendix 1). Participants who answered “no” or “uncertain” were directed to a specific module to rate 9 distinct fears (question 13 in the Multimedia Appendix 1). These 9 items captured specific implementation barriers across several theoretical domains, including technological rigidity, regulatory gaps, and data governance from the NASSS framework, as well as perceived effectiveness, affective attitudes, and ethicality from the Theoretical Framework of Acceptability. To capture precise differences in patient anxiety, we used a granular scale from 0 (not at all concerned) to 10 (extremely concerned). For a clear descriptive analysis, we grouped these scores into 3 levels: 0 to 3, low concern; 4 to 6, moderate concern; and 7 to 10, high concern.

In addition, the questionnaire assessed participants’ familiarity with and experience of AI, including self-reported technical knowledge, general familiarity, and perceived prior exposure to AI-based diagnostic tools. Finally, sociodemographic and clinical characteristics, such as age, education, employment status, prior health care experience, and trust in the health care system, were collected to contextualize participants’ responses, as these factors have been shown to influence perceptions and acceptance of health care technologies [7,9].

Data collection for the quantitative phase took place from January to December 2025. The questionnaire was initiated 99 times via the provided link, yielding 51 fully completed responses; incomplete entries due to technical errors or withdrawal were automatically excluded via REDCap, and only fully completed questionnaires were retained for analysis.

#### Data Analysis

Categorical and ordinal variables were summarized using frequencies and percentages. Key variables included perceived benefits of AI, willingness to use AI-based diagnostic tools (yes/no/uncertain), and concerns among participants who were unsure or unwilling to use AI. Given the limited sample size and the distribution of responses, analyses were restricted to descriptive summaries. Quantitative analyses were conducted using R software (version 4.5.0; R Foundation for Statistical Computing).

### Qualitative Phase

#### Participants and Recruitment

EK recruited patients via phone from those who had consented to participate following the quantitative survey. For the patient sample (N_1_=16), she contacted 24 patients who had previously agreed to be interviewed and had provided their telephone numbers in the survey. Of the 24 patients contacted, 16 (67%) participated. The remaining patients declined participation, were unavailable at the proposed times, or could not be reached. Due to the patients’ varying time availability, we scheduled the sessions flexibly. EK also recruited physicians from 2 sources (N_2_=11), using a purposive sampling strategy to identify participants with clinical expertise relevant to prostate cancer diagnosis and care. Fifteen physicians from CHU de Liège were invited via email, of whom 7 (47%) agreed to participate. In addition, an invitation was distributed through the Belgian Society of Radiology, and 4 radiologists who responded to the invitation were subsequently contacted and included in the study. Physicians were eligible if they were actively involved in prostate cancer diagnosis or treatment and had at least 1 year of clinical experience in their specialty.

Participants received a fixed compensation to cover participation-related expenses (eg, travel costs).

#### Data Collection

Data were collected through semistructured focus groups and individual interviews, using guides developed from the study objectives and informed by the quantitative findings. In particular, survey results highlighting perceived benefits of AI, divided willingness to use AI-based tools, and recurring concerns (eg, responsibility, loss of human interaction, and data confidentiality) were used to refine and deepen the qualitative exploration of these issues.

During the focus groups and interviews, the sessions began with a standardized introductory explanation of the future FLUTE platform. Participants were asked to consider an anticipated scenario where the AI tool would act alongside physicians to improve magnetic resonance imaging interpretation and help safely avoid unnecessary biopsies. The guides explored participants’ care pathways, perceptions of the benefits and risks of AI, its impact on communication and decision-making, and conditions for acceptable implementation. For physicians, additional attention was given to their experiences with AI, their role in informing patients, perceptions of patient concerns, and conditions required for its integration into clinical practice. Probing questions were used to explore these topics in depth. The full interview guides are provided in Multimedia Appendices 2 and 3.

With patients, we conducted 4 focus groups and 4 individual semistructured interviews. The focus groups were organized dynamically based on when patients were available: 2 groups consisted of 4 participants each, and 2 groups consisted of 2 participants each. For the physician sample, 11 individual semistructured interviews were conducted. Data collection continued until the dataset was considered sufficiently rich, diverse, and relevant to support a meaningful analysis of the research questions.

Interviews were conducted either in person or remotely via Microsoft Teams, using an institutional digital audio recorder or the secure Teams environment to ensure data privacy. To maintain data security, raw audio files were transcribed using an offline, locally installed deployment of Whisper (OpenAI), avoiding external cloud servers. EK then reviewed all transcripts against the original audio to ensure verbatim accuracy and eliminate AI errors. All direct identifiers were systematically removed from the text and replaced with unique alphanumeric codes constructed using a strict formula based on the first and last letters of the surname, the first letter of the first name, and the last 2 digits of the birth year. The master key linking these codes to real identities was stored separately on an encrypted university server.

Focus groups lasted approximately 90 minutes, while interviews lasted between 30 and 60 minutes. Interviews with physicians were conducted in French or English by EK, who had received formal training in qualitative research methods during her master’s degree in psychology. EK also conducted 2 individual patient interviews. Patient focus groups were conducted in French and moderated by DK, who holds a master’s degree in public health sciences. EK was present during the focus groups as a nonparticipant observer and supported facilitation when necessary. Both researchers were familiar with the study topic and contributed to the development of the interview guides. Neither researcher had a prior clinical relationship with the participants.

The qualitative phase was conducted between October and December 2025.

#### Data Analysis

Qualitative data management, organization, and manual coding were performed using NVivo software (version 15; Lumivero), without the use of any automated or AI-based coding features.

The data were analyzed collaboratively by 3 researchers (EK, DK, and BV) using an iterative, inductive thematic analysis. We followed the principles of reflexive thematic analysis [31,32], which conceptualizes the researcher not as a neutral instrument but as an active, subjective coproducer of meaning. To enrich the interpretive depth and capture nuanced patterns, EK independently coded the entire dataset, while DK and BV coded half of the transcripts each. The analysis was initially guided by the study focus and quantitative findings, which highlighted perceived benefits of AI as well as concerns related to responsibility, communication, and decision-making. In the first stage, we analyzed transcripts using descriptive coding focused on participants’ perceptions and experiences regarding the integration of AI into prostate cancer diagnostics. Through this initial coding process, uncertainty emerged as a central pattern across participants’ accounts. We therefore conducted a subsequent, more focused analysis using uncertainty as an analytical lens to examine how it was expressed and negotiated in relation to AI-supported diagnostics. This analysis led to the identification of three interrelated dimensions of uncertainty: (1) epistemic uncertainty, concerning the interpretation and reliability of AI-generated knowledge; (2) ethical uncertainty, related to the allocation of responsibility for AI-informed decisions and potential errors; and (3) role-related uncertainty, concerning changing roles, communication, and decision-making between patients and physicians.

#### Reflexivity and Team Collaboration

Within these dimensions, we worked collaboratively—not to achieve a standardized consensus but to reflexively deepen and enrich our interpretations of the developing subthemes. Rather than seeking a positivist interrater reliability checking or strict code consensus, we embraced our diverse research backgrounds (psychology, public health, and sociology) as an active analytical tool. This collaborative approach allowed us to capture different layers of meaning, including the psychological nuances of individual anxiety and trust, the public health implications for patient-centered workflows, and the broader sociological shifts in professional roles. We ensured the trustworthiness and credibility of our findings through continuous team reflexivity, deep immersion in the transcripts, and grounding every theme in the participants’ actual words.

Where appropriate, individual quotations were cross-coded with multiple codes to capture overlapping meanings, and this dynamic structure resulted in the final thematic framework, which was applied across all transcripts.

Crucial to this collaborative reflexivity was the recognition of our own sociodemographic and generational positioning. As a research team younger than most participants, we naturally had higher technical familiarity and a more intuitive acceptance of AI. To ensure that this digital optimism did not cloud our interpretation, we used continuous team discussions to actively challenge our assumptions. This allowed us to carefully preserve the unique hesitations and traditional trust values expressed by the older patients and physicians.

To present the findings in English, all participant quotations originally stated in French were translated by EK, who also holds a university degree in linguistics, and double-checked by DK and BV to ensure that the exact clinical and psychological meanings were fully preserved.

## Results

### Quantitative Phase

#### Sample Characteristics: Familiarity With AI and Prior Use of AI Tools

A total of 51 patients were included in the study, with a mean age of 66.96 (SD 7.39, range 51‐82) years. Sample characteristics, including AI-related background and health care–related perceptions, are summarized in Table 2.

**Table 2.** Participant characteristics, prior AI exposure, and health care–related perceptions (N=51).

Variable	Values
Age (years)	
Mean (SD)	66.96 (7.39)
Range	51‐82
Employment status, n (%)	
Retired	38 (74.5)
Employed full time	12 (23.5)
Unemployed	1 (2)
Education level, n (%)	
Primary education	1 (2)
Secondary education	18 (35.3)
Nonuniversity higher education	23 (45.1)
University degree	6 (11.8)
PhD	3 (5.9)
Prior use of AI in diagnostics, n (%)	
Yes	4 (7.8)
No	35 (68.6)
Not sure	12 (23.5)
General familiarity with AI, n (%)	
Not at all familiar	9 (17.6)
Not familiar	16 (31.4)
Neutral	16 (31.4)
Familiar	8 (15.7)
Very familiar	2 (3.9)
Self-assessed technical knowledge of AI, n (%)	
Very poor	1 (2)
Poor	19 (37.3)
Average	21 (41.2)
Good	9 (17.6)
Excellent	1 (2)
Previous health care experience, n (%)	
Very poor	1 (2)
Poor	3 (5.9)
Average	1 (2)
Good	32 (62.7)
Very good	14 (27.5)
Trust in health care institutions, n (%)	
Best quality of care	
Strongly disagree	1 (2)
Disagree	0 (0)
Neither agree nor disagree	6 (11.8)
Agree	37 (72.5)
Strongly agree	7 (13.7)
Patients’ needs above costs	
Strongly disagree	1 (2)
Disagree	2 (3.9)
Neither agree nor disagree	9 (17.6)
Agree	36 (70.6)
Strongly agree	3 (5.9)

As presented in the table, prior experience with AI-based health care technologies was limited. The majority of participants reported that AI had not been used in the diagnostic process leading to their cancer diagnosis, while only a small proportion indicated that it had been used. A notable proportion of respondents were unsure whether AI had been involved in their care.

Familiarity with AI was predominantly low to moderate. Nearly half of the sample described themselves as not at all or not familiar with AI, whereas only a small minority reported high familiarity. Self-reported technical knowledge followed a similar pattern, with most participants rating their level of understanding as average or poor and very few indicating advanced knowledge.

Furthermore, although participants reported limited familiarity with AI and little prior exposure to AI-based diagnostic tools, their general orientation toward health care appeared to be largely positive. Previous health care experience was evaluated favorably, with 90.2% (n=46) of respondents rating it as good or very good. Trust in health care institutions was also generally high: most participants agreed that health care institutions provide the best quality of medical care (n=44, 86.2%) and that they place patients’ medical needs above other considerations, including costs (n=39, 76.5%). Overall, these findings indicate that limited familiarity with AI in this sample occurred in the context of generally positive prior health care experiences and relatively high trust in health care institutions.

#### Perceived Benefits of AI in Prostate Cancer Diagnosis

Participants evaluated the potential benefits of AI-based tools in prostate cancer diagnosis. As shown in Figure 1, their perceptions of potential benefits were predominantly positive. Most respondents agreed or strongly agreed that AI can provide an accurate diagnosis (n=32, 62.7%), improve prostate cancer diagnosis overall (n=36, 70.6%), and advance the diagnostic process (n=35, 68.6%). Across all benefit-related items, explicit disagreement was uncommon.

**Figure 1.**
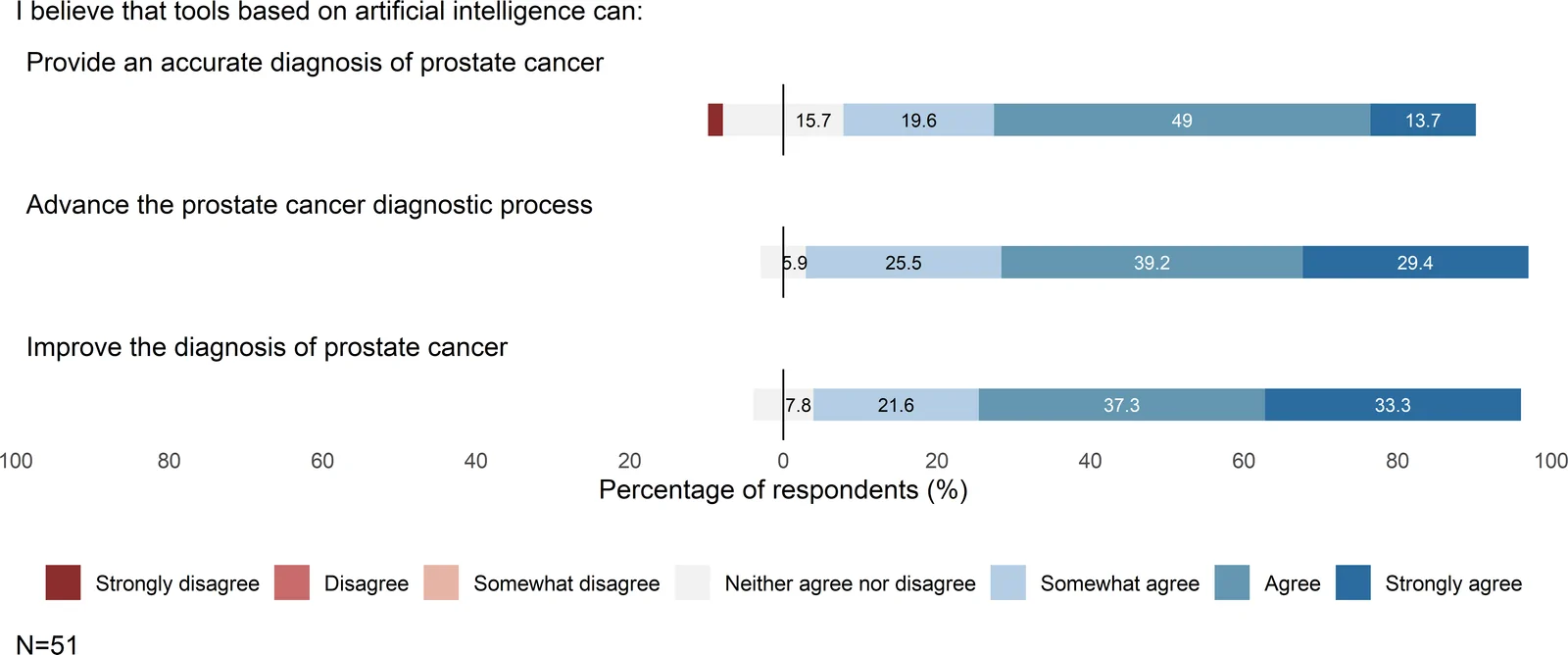
Perceived benefits of AI-based tools in prostate cancer diagnosis (N=51). Bars represent the percentage distribution of responses across agreement levels (strongly disagree to strongly agree).

Although perceptions of AI-related benefits were predominantly positive, responses became more differentiated when participants were asked about their own willingness to use AI-based tools in prostate cancer diagnosis. As shown in Figure 2, approximately half (23/51, 45.1%) of the participants indicated that they would like to use AI-based diagnostic tools, whereas a similarly large proportion remained uncertain, and some respondents stated that they would not want to use such tools. These findings suggest that positive perceptions of AI benefits were accompanied by considerable hesitation regarding personal use.

**Figure 2.**
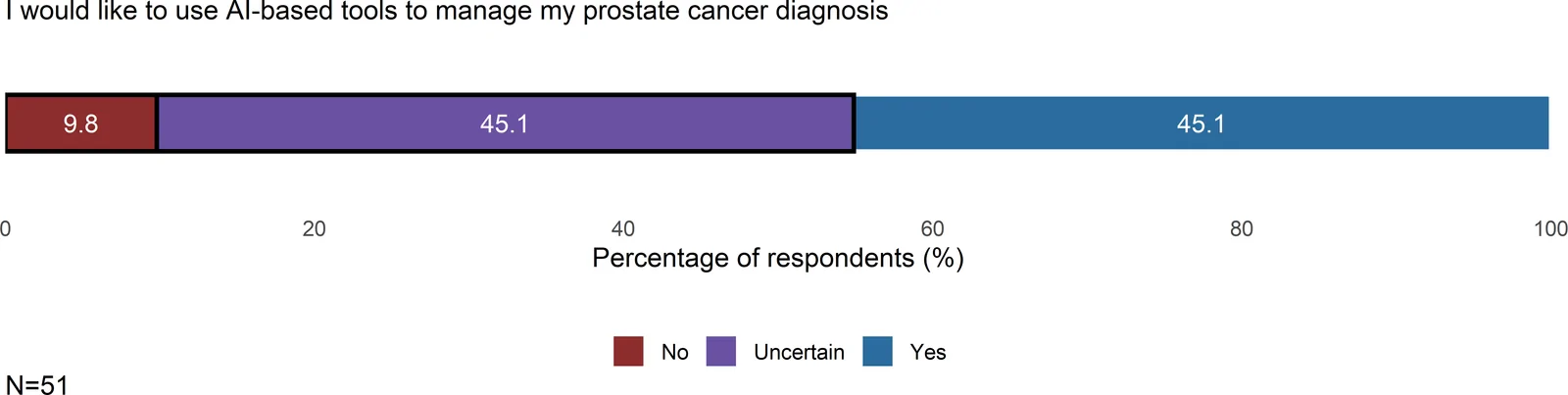
Intention to use AI-based tools in prostate cancer diagnosis (N=51). Bars represent the percentage distribution of responses (no, uncertain, and yes).

Participants who were uncertain or unwilling to use AI-based tools (n=28, 0.55 %) were additionally asked to evaluate a set of potential concerns related to their use. Concerns varied across domains, with issues related to loss of human connection and accountability more often rated as highly concerning than other dimensions (Figure 3). In contrast, issues such as inaccurate diagnosis, poor adaptability, and general lack of trust in AI were more frequently evaluated as moderate rather than high concerns. Privacy-related issues and unclear regulation also emerged as relevant concerns, although these were typically rated in the low to moderate range rather than at the highest level of concern.

**Figure 3.**
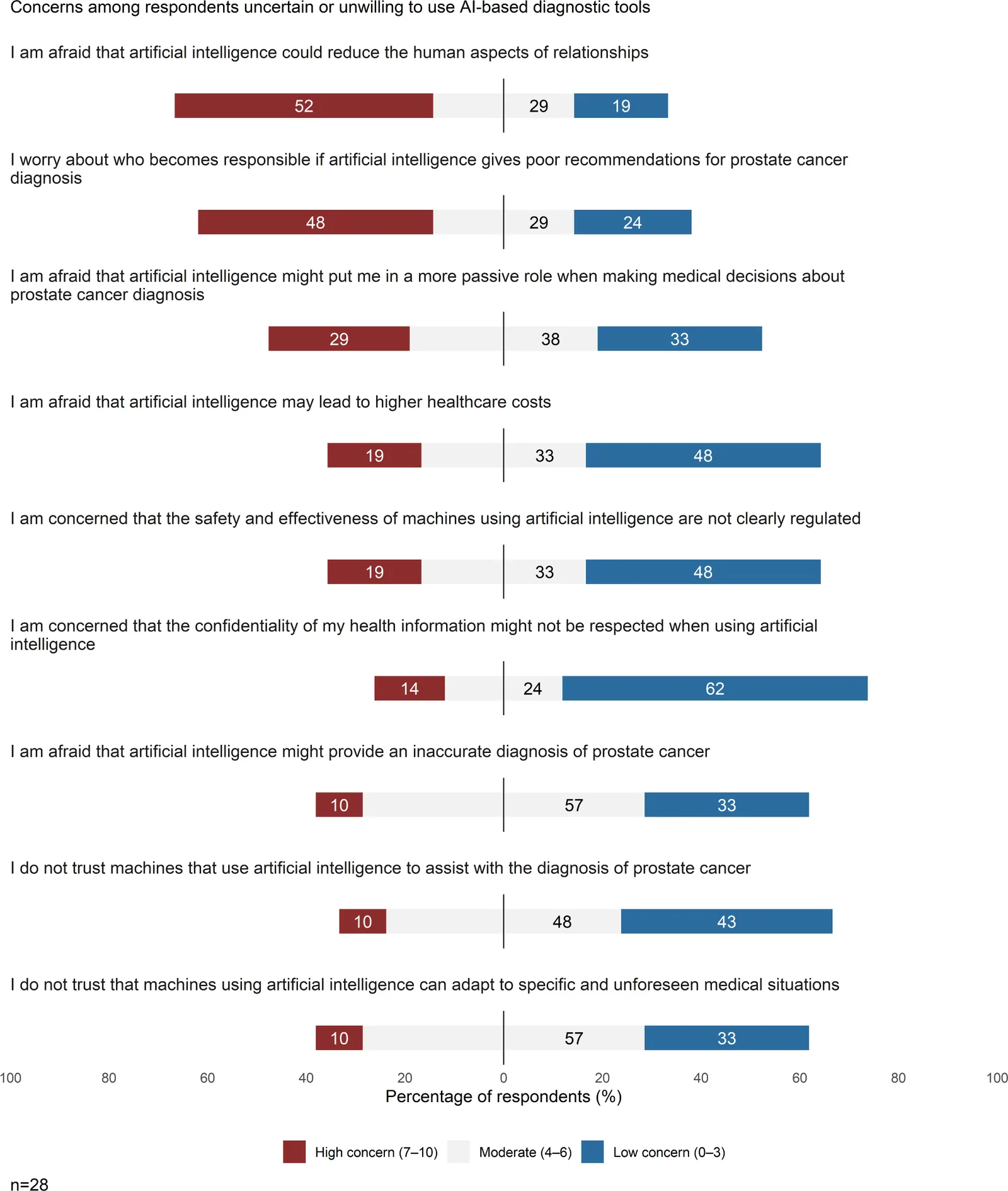
Reported concerns regarding the use of AI-based diagnostic tools among participants who were unsure or unwilling to use them (n=28). Bars represent the percentage distribution of concern levels across domains.

These findings were further explored in the qualitative phase.

### Qualitative Phase

#### Sample Characteristics: Patients and Physicians

A total of 27 participants participated in the qualitative phase, including 16 (59%) patients and 11 (41%) physicians (radiologists: n=5, 45%; urologists: n=4, 37%; and radiation oncologists: n=2, 18%). Patients reflected a range of educational and employment backgrounds, broadly comparable to the quantitative sample. Physicians represented different clinical roles within prostate cancer diagnostics and had varying levels of professional experience, reporting generally higher levels of familiarity with AI alongside comparatively lower self-assessed technical knowledge. The demographic and professional characteristics of participants are summarized in Table 3.

**Table 3.** Qualitative sample characteristics.

Variable	Values
Patients (N_1_=16)	
Age (years)	
Mean (SD)	65.0 (6.4)
Range	51‐75
Employment status, n (%)	
Retired	11 (68.8)
Employed	5 (31.3)
Education level, n (%)	
Primary education	2 (12.5)
Secondary education	4 (25)
Nonuniversity higher education	7 (43.8)
University degree	2 (12.5)
PhD	1 (6.3)
Prior use of AI in health care, n (%)	
Yes	3 (18.8)
No	7 (43.8)
Not sure	6 (37.5)
General familiarity with AI, n (%)	
Very familiar	0 (0)
Familiar	7 (43.8)
Neutral	3 (18.8)
Not familiar	5 (31.3)
Completely not familiar	1 (6.3)
Self-assessed technical knowledge of AI, n (%)	
Excellent	0 (0)
Good	4 (25)
Average	6 (37.5)
Poor	5 (31.3)
Very poor	1 (6.3)
Physicians (N_2_=11)	
Gender, n (%)	
Male	8 (72.7)
Female	3 (27.3)
Specialization, n (%)	
Radiology	5 (45.5)
Urology	4 (36.4)
Radiation oncology	2 (18.2)
Prior use of AI in practice, n (%)	
Yes	7 (63.6)
No	4 (36.4)
General familiarity with AI, n (%)	
Very familiar	2 (18.2)
Familiar	4 (36.4)
Neutral	3 (27.3)
Not familiar	1 (9.1)
Completely not familiar	1 (9.1)
Self-assessed technical knowledge of AI, n (%)	
Excellent	1 (9.1)
Good	2 (18.2)
Average	4 (36.3)
Poor	3 (27.3)
Very poor	1 (9.1)

#### Thematic Analysis Findings

##### Findings Overview

Three dimensions corresponding to different ways in which AI may destabilize the implicit certainties structuring the patient-physician relationship emerged from the analysis: epistemic uncertainty, ethical uncertainty, and uncertainty of social roles. Epistemic uncertainty concerns the status and interpretation of AI-generated knowledge; ethical uncertainty refers to questions of responsibility and legitimacy; and uncertainty of social roles addresses the reconfiguration of roles between patients, physicians, and technological systems.

The following sections present the results of the qualitative analysis according to these analytical dimensions. An overview of these results is also summarized in Figure 4.

**Figure 4.**
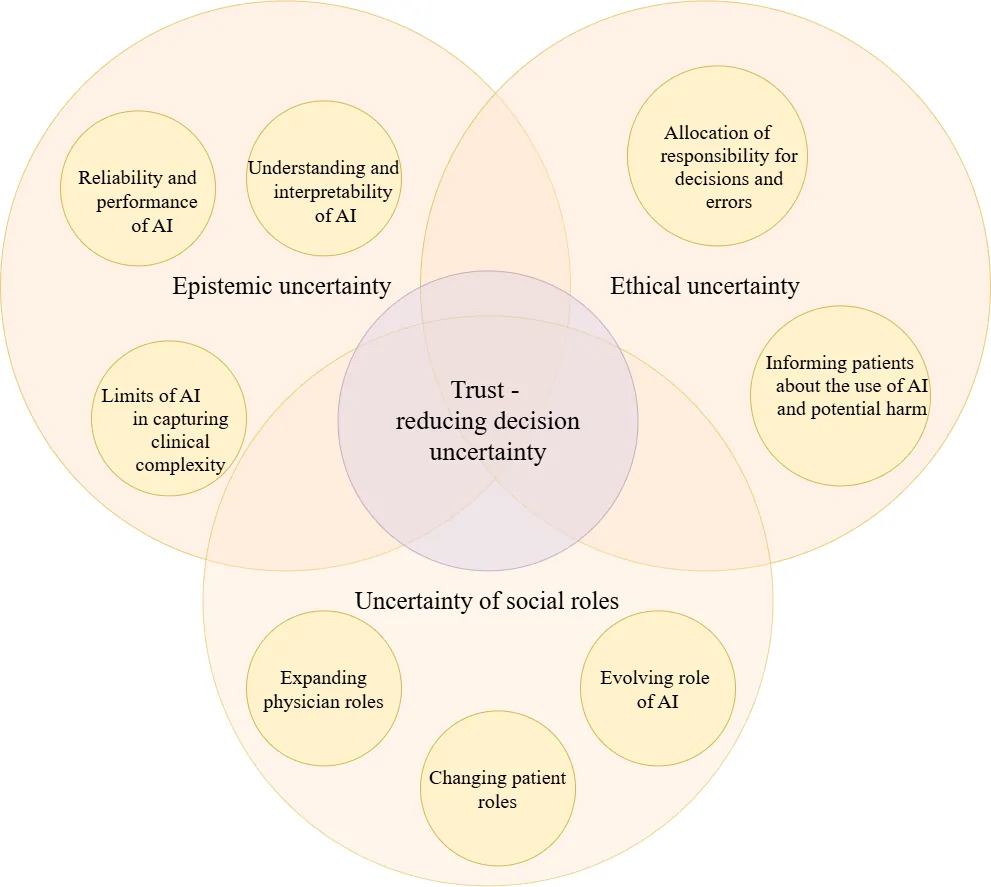
Thematic tree linking trust in medical decision-making with epistemic, ethical, and social role uncertainties. The central trust construct is not separate from these domains; instead, it is directly connected to how patients and physicians negotiate gaps in AI knowledge, accountability for errors, and changing clinical roles.

##### Epistemic Uncertainty

###### Overview

Participants described several uncertainties related to AI-generated information, including how it could be understood and interpreted, how its performance could be evaluated in practice, and how it could be integrated into the broader clinical context.

###### Understanding and Interpretability of AI

Among patients, epistemic uncertainty centered primarily on difficulties in understanding how AI systems work and how their outputs can be interpreted, rather than on their accuracy. Patients questioned how AI processes information, where its data come from, and how they are selected, combined, and maintained. Questions about who stands behind these systems and who is responsible for providing and curating the data were frequent, and lack of clarity made AI-generated information difficult for some patients to understand and evaluate:

*No, what are the sources of information and how are they compiled? How can we say, with artificial intelligence, that we have enough information? And where does the information come from? Who inputs this information to make it realistic? That’s something I still can’t quite grasp.*

[Patient 8, focus group P3]

Patients also highlighted the importance of clinical interpretation of the AI-generated information. Patients therefore emphasized the need for AI-generated information to be explained and contextualized by a health care professional:

*I don’t always clearly understand how they manage to process all that information, or what kind of information they use to feed the system and arrive at a diagnosis.*

[Patient 9, focus group P3]

###### Reliability and Performance of AI in Clinical Practice

In contrast to patients, physicians primarily framed epistemic uncertainty in terms of the reliability and clinical performance of AI-generated knowledge. They did not take AI outputs at face value and emphasized the need to assess systems through established metrics such as accuracy, error rates, false positives, negative predictive value, and reproducibility. A recurring concern was the need for proper scientific validation, including clinical evaluation and performance studies, before integrating AI into routine practice. Uncertainty was also linked to the data used to train these systems, with physicians pointing to potential issues of data quality, representativeness, and bias, as well as a lack of transparency regarding how models are developed.

In addition to concerns about data quality and validation, physicians also raised issues related to how AI systems behave in real clinical situations. A recurring point was that AI tends to always produce an answer, even in cases where physicians would normally remain uncertain or consider multiple possibilities, potentially creating an impression of certainty that is not always justified:

*The software will never say that it doesn’t know. It will always provide an answer.*

[Radiologist, interview D7]

A related but distinct concern was the tendency of AI systems to prioritize sensitivity over specificity. This can result in a higher number of false positives, confusing diagnosis, prompting additional investigations, and potentially affecting trust, particularly when such results are visible to patients without sufficient explanation:

*...probably the AI will detect, let’s say, every cancer, but of course not everything it highlights is cancer.*

[Radiologist, interview D6]

Interestingly, patients highlighted the potential usefulness of AI in “complex cases,” while physicians emphasized its value in “gray zones” of diagnostic uncertainty. However, these are precisely the situations in which physicians currently have the lowest confidence in its outputs, highlighting a gap between the contexts in which AI is expected to be most useful and those in which it is perceived as most reliable.

###### Limits of AI in Capturing Clinical Complexity

More broadly, physicians insisted that medical knowledge cannot be reduced to algorithmic processing alone. Clinical interpretation was described as involving context, experience, and judgment—elements that are not easily captured by AI and are often seen as remaining within the domain of human physicians.

As one physician explained, clinical decision-making relies not only on formal guidelines but also on a “personal algorithm” shaped by experience and by the specific patient-physician relationship, making it unclear “where AI is going to help”

*One patient is not the same as another [...] their age, their comorbidities [...] their psychology [...] that belongs to the human domain.*

[Urologist, interview D10]

In particular, psychological aspects of patient care were highlighted as beyond the reach of AI. Both physicians and patients stressed that clinical decisions involve an intuitive understanding of the patient—including their psychological state—which was seen as part of human judgment and not easily reducible to formalized or computational processes. Patients particularly emphasized the importance of the relational dimensions in clinical care.

### Ethical Uncertainty

#### Overview

Ethical uncertainty was primarily related to questions of responsibility and whether patients should be informed about the use of AI. In contrast to epistemic uncertainty, the focus here was not on the quality of knowledge, but on the consequences of its use for the patient-physician relationship and for decision-making.

#### Allocation of Responsibility for Decisions and Errors

Patients focused strongly on questions of responsibility, emphasizing the need for a clearly identifiable accountable actor. They consistently stated that medical decisions should remain the responsibility of a physician, with AI used only as a supporting tool under human control:

*Responsibility must not be left unclear. There has to be a physician behind it.*

[Patient 2, focus group P2]

Physicians expressed a similar view, consistently stating that responsibility ultimately remains with them. At the same time, they pointed to a tension: although they are expected to take responsibility, they do not control how AI systems are designed or how they function, echoing earlier concerns about the opacity of these systems. This creates a situation in which physicians remain accountable for outcomes, including potential errors, without having full control over the tools they use. One physician noted that it would be preferable for developers to share responsibility but also acknowledged that this is unlikely in practice:

*If we still have to supervise everything ourselves and the responsibility is still with us, then it’s not worth the money. Actually, it would be better if [...] they [developers] would take the responsibility.*

[Radiologist, interview D9]

This tension was also reflected in how participants discussed potential errors. Patients were more accepting of errors made by physicians than of errors attributed to AI. Across their accounts, this preference appeared alongside an emphasis on the physician as an identifiable person who could remain responsible and provide explanations.

#### Informing Patients About the Use of AI and Potential Harm

From the patients’ perspective, being informed about the use of AI was not seen as necessary in all cases. For many, information about AI only became relevant when they needed to better understand their condition or discuss their care. AI was often perceived as part of the physician’s internal process, rather than something that needed to be explicitly disclosed:

*There’s no real benefit in me knowing that. [...] It’s his tool, he doesn’t need to tell me.*

[Patient 3, focus group P2]

At the same time, views varied depending on patients’ personal attitudes toward control and trust. For some, trust in the physician reduced the perceived need for detailed information about the tools used, while for others, a stronger need for control was associated with a preference for being more fully informed:

*If a plumber comes to my house, I don’t ask what tool he uses. I don’t care. [...] I trust the physician.*

[Patient 3, focus group P2]

*I would prefer to know. [...] I would prefer him to tell me, because then I know that it is the physician’s own opinion, but that his opinion has also been influenced by artificial intelligence.*

[Patient 15, interview P7]

Physicians expressed more divided views on this issue. Some considered informing patients about the use of AI as part of respecting patient autonomy, particularly when AI contributes to decision-making. Others questioned the value of disclosure, arguing that patients are primarily concerned with outcomes and that mentioning AI may create confusion:

*In general, we communicate a main line of reasoning to the patient [...] we don’t mention all the discussions [...] that could be seen as unsettling.*

[Urologist, interview D10]

*I’m afraid it would add confusion to the clarity of our report.*

[Radiologist, interview D4]

Several physicians also linked disclosure to potential harm. AI systems designed to minimize missed diagnoses may generate more false positives, exposing patients to findings that are clinically uncertain or irrelevant. Making such information visible to patients was seen as potentially leading to unnecessary anxiety, undermining trust in clinical judgment, and prompting additional interventions, especially when AI outputs conflict with their own assessment.

### Uncertainty of Social Roles

#### Overview

The epistemic and ethical uncertainties described earlier were also reflected in discussions about the distribution of roles in clinical practice. Participants discussed possible changes in how physicians and patients would use information, make decisions, and distribute responsibilities when AI is introduced. We interpreted these accounts as reflecting uncertainty about the evolving social roles of physicians, patients, and other actors in decision-making.

#### The Evolving Role of AI: Can It Shift the Position of Physicians?

Clinical care was widely described as involving more than technical diagnosis. Patients highlighted the importance of explanation, reassurance, and human support, particularly in situations of clinical complexity, where physicians are expected to interpret findings, adapt them to individual situations, and provide emotional guidance:

*For me, it should remain a tool that supports the physician, so you would always have a doctor in front of you who explains things to you.*

[Patient 1, interview P1]

*People, if they don’t have someone behind them to hold their hand, to calmly explain to them that it may not be serious, that it may be serious [...], we are going to lose that [...] and people, they need, really need human, psychological support, of quality, in particular in cancer.*

[Patient 15, interview P7]

Physicians expressed similar views, emphasizing that their role cannot be reduced to technical tasks. Even where AI was seen as capable of supporting analytical functions, clinical care was described as involving communicative and relational work that cannot be delegated in the same way:

*It is necessary to explain to the patient that he has cancer but that it is not treated because it is not dangerous to monitor it.*

[Urologist, interview D10]

In this context, AI was often described as a form of “tool,” providing an additional input rather than functioning as an actor equivalent to human expertise. While the possibility of partial substitution was occasionally mentioned—particularly in fields such as radiology—the complete replacement of physicians was considered unlikely:

*I do not think that artificial intelligence will replace a physician—I sincerely hope it never will.*

[Urologist, interview D1]

Discussions also pointed to differences in experience, with more experienced practitioners seen as less replaceable, while younger or less experienced practitioners who learn with AI may become more dependent on it and may develop weaker independent skills

*Young radiologists who learn radiology with the software, I am not sure they have as good a knowledge as those of us who learned without it. And if tomorrow they no longer have it, I think they will be in difficulty.*

[Radiologist, interview D7]

#### Expanding Physician Roles: Interpretation, Oversight, and Participation in AI Development

While patients often associated AI with technological progress and greater efficiency, physicians—particularly radiologists—described its introduction as a source of pressure. Adoption was often perceived as increasingly inevitable, yet AI was not always seen as clearly improving practice. Instead, some physicians described it as adding complexity without sufficient added value, particularly given earlier concerns about oversensitivity:

*We have some pressure, and we could say, as a group here, that we don’t need it; but if you don’t use it, there may be a feeling that we could miss the boat.*

[Radiologist, interview D6]

This pressure was closely linked to an expansion of physicians’ roles. Beyond their clinical responsibilities, they were increasingly expected to supervise and monitor AI systems, interpret their outputs, and engage in their evaluation and development:

*We have to stay active as medical doctors in this domain because I think we are the ones that should steer the direction in which AI is being trained and developed [...] medical doctors should also contribute in the development of AI.*

[Radiologist, interview D5]

These expectations were reinforced by external pressures related to the broader implementation of AI, including expectations of use, commercial interests, and concerns about being evaluated against system outputs. As a result, physicians described the integration of AI as extending their professional activities without a corresponding reduction or redistribution of workload, contributing to a sense of increased responsibility and scrutiny:

*It does not help us to reduce the time for looking at the scans. So, we just look at the scans the same way we do it [...] it does not help us in a time perspective.*

[Radiologist, interview D6]

*If we still have to supervise everything ourselves and the responsibility is still with us, then it’s not worth the money.*

[Radiologist, interview D9]

*The problem is that there are obviously commercial stakes, because these software tools are developed, they are produced, and then they are sold by people who will increasingly and continuously try to impose these tools on us and make us dependent on them, and I think we still need to be quite vigilant.*

[Radiologist, interview D7]

This expansion also extended beyond the immediate clinical encounter. Some physicians described AI implementation as a collective and organizational process requiring multidisciplinary coordination, institutional support, and involvement from hospitals, technical teams, and decision-making bodies. In this sense, physicians were not only expected to use and supervise AI tools but also to contribute to the conditions under which these tools are adopted and integrated into practice.

*You need to define a whole team of people, multidisciplinary, who can make a strategy... The hospital should be open for this and they should be willing to invest in this and they should understand. And when I say the hospital, then I speak about the medical boards and I also speak about the board of directors.*

[Radiologist, interview D5]

#### Changing Patient Roles: Tensions Between Autonomy and Dependence

The introduction of AI appeared to reshape the patient’s role in an ambivalent way: increased access to information encourages more active engagement for some patients, while reinforcing dependence on the physician for others. Some participants associated information about AI use with a desire to better understand their situation and retain a degree of control over decisions:

*I think that the patient or the patients must be informed about the impact of AI at the medical level if it is used in that sense... so that the patient can say, well no, I don’t have trust. Yes, I have trust. Can decide in terms of an intervention or not, depending on whether AI comes into play or not.*

[Patient 5, focus group P2]

*I am not someone who is anxious a priori, but I like to have the information and to compile it by myself. And also to have information coming from someone else, to form an idea, to know where I stand. And that reassures me personally.*

[Patient 6, focus group P3]

Physicians also emphasized that patients are not equally prepared to engage with complex information: while some actively seek it out, others may feel confused or overwhelmed, especially when faced with multiple possible options:

*Sometimes there are two equally valid options, and when patients are asked to choose, they can feel completely lost.*

[Radiation oncologist, interview D2]

At the same time, both patients and physicians pointed to important limits to a shift in patients’ position. Participants suggested that access to more information did not always make decision-making easier, particularly when information was complex or ambiguous.

To visually summarize the integration of our data, Table 1 presents a joint display that maps how the initial quantitative survey benchmarks informed the subsequent qualitative inquiry and how the multistakeholder qualitative insights explain and extend the statistical trends.

## Discussion

### Principal Results

Our mixed methods study shows that attitudes toward AI in prostate cancer diagnostics cannot be reduced to a simple opposition between acceptance and rejection. Quantitatively, patients tended to view AI as potentially beneficial for diagnostic accuracy and efficiency, yet this did not consistently translate into a willingness to use AI-based tools in their own diagnostic pathway. Qualitatively, this hesitation can be explained through the lens of uncertainty: participants assess AI not only in terms of performance but also in relation to trust, responsibility, communication, and the potential redistribution of roles in clinical practice. Taken together, these findings suggest that attitudes toward the integration of AI in prostate cancer diagnostics depend not only on perceived technological benefits but also on how AI is situated within existing relationships of trust, responsibility, and communication in clinical care.

### Comparison With Prior Work

A key finding of the quantitative phase is the tension between perceived utility and personal willingness to use AI. While many participants recognized the potential benefits of AI in prostate cancer diagnostics, their willingness to use such systems remained divided, with hesitancy being more common than clear endorsement. Importantly, relatively low levels of prior exposure, familiarity, and technical confidence in our sample may help explain these cautious attitudes. With many participants unsure whether AI was already involved in their care, evaluations were often formed in the absence of direct experience. At the same time, these perceptions were shaped by positive previous health care experience and generally high baseline trust in health care institutions. Within this context, AI was perceived as a nontransparent element introduced into an otherwise trusted system, suggesting that existing trust in physicians and health care institutions may play an important role in how patients approach clinical AI [22].

This pattern is particularly relevant in the context of prostate cancer diagnostics, which has been widely described as a paradigmatic case for shared decision-making due to the need to balance risks and benefits [24]. In this setting, decision-making is inherently shaped by clinical complexity. While patient-centered care promotes autonomy and active participation, it also exposes patients more directly to complex, probabilistic, and sometimes ambiguous information that is traditionally managed by clinicians [33-35], a dynamic that may be intensified by the integration of AI.

Against this background, recent works have highlighted that AI systems introduce specific forms of technical challenges related to model performance, interpretability, and the handling of complex clinical data [36,37]. However, our findings suggest that the impact of AI cannot be reduced to these technical aspects alone. Rather than reducing information complexity, the introduction of AI appears to transform how uncertainty is experienced and managed, particularly by reshaping how knowledge is interpreted, how responsibility is assigned, and how roles are negotiated between patients, physicians, and technological systems. In this context, trust remains a central condition for decision-making, mediating how these transformations are accepted, negotiated, or resisted in practice.

Epistemically, patients primarily focused on the question of whether AI-generated information can be understood and trusted. Their concerns centered on where the information comes from, who stands behind it, and whether it can be made meaningful in the context of their own care. These findings are consistent with previous research showing that patients’ trust in AI depends on its intelligibility and on physician-mediated interpretation, particularly when the system remains difficult to understand [12,22,38]. This is also supported by evidence from prostate cancer care, where trust in AI remains closely tied to the treating clinician and the clinical context [20,39].

Physicians, in contrast, questioned the reliability, validation, reproducibility, and clinical relevance of AI outputs, especially in ambiguous cases. Their concerns align with existing literature, indicating that clinicians’ confidence in AI depends on its performance, robustness, and integration into clinical workflows [8,40], while uncertainties persist regarding validation methods and real-world applicability in prostate cancer diagnostics [16]. Furthermore, our findings suggest that physicians’ trust in AI was particularly challenged in the “gray zones” of prostate cancer diagnostics, where clinical interpretation was already characterized by uncertainty. Physicians did not reject AI per se but questioned its ability to handle context appropriately, particularly when it generated false positives or noncontributory signals. These concerns align with literature showing that trust in clinical decision support systems is weakened when systems generate excessive or clinically irrelevant alerts, leading to alert fatigue and increased cognitive burden [41]. They also resonate with broader evidence that poorly integrated systems may disrupt workflows and fail to provide meaningful added value, thereby limiting their effective use in practice [42]. In addition, concerns about bias in AI systems—particularly in underrepresented patient groups—raise further questions about the reliability and fairness of AI-supported decisions [43].

Taken together, these epistemic limitations point to a broader issue noted by both patients and physicians: AI systems often struggle to account for the full clinical context, which involves not only imaging findings but also patient characteristics, psychological factors, comorbidities, and experiential judgment [2,34]. As a result, AI-generated outputs may remain difficult to interpret in isolation and require clinical contextualization to become meaningful in practice [44,45].

These epistemic concerns are closely linked to ethical uncertainty because questions about the reliability and interpretation of AI outputs also raise questions about who should be accountable for their use in clinical decision-making. Ethical uncertainty in our findings is primarily structured around questions of responsibility, error, and disclosure.

One ethical aspect that emerged was responsibility. Patients consistently emphasized the need for a clearly identifiable human decision-maker, and physicians maintained that accountability ultimately remains with them, even when they do not fully control how AI systems are developed or function. While existing literature often conceptualizes responsibility in AI-supported care as distributed across multiple actors, including physicians, health care institutions, and developers [46,47], our findings reflect a more physician-centered framing. This may partly relate to how responsibility was operationalized in our study, but also suggests that, in practice, participants anchor accountability in the clinician as the most immediate and answerable actor.

This physician-centered framing helps explain why support for AI remained conditional in our material: AI was acceptable when it assisted physicians without displacing human judgment and when physician oversight and accountability were maintained. This aligns with prostate-specific studies showing that patients are open to AI involvement but prefer it to remain embedded within physician-led care [20,21], as well as with broader evidence that acceptance is higher when AI is framed as supportive rather than autonomous [38,39,48].

Furthermore, this distribution of responsibility gives rise to an important asymmetry. Physicians remain accountable for decisions while relying on systems they do not fully control, a tension widely noted in the literature on AI in health care [43,49]. This tension becomes particularly salient in situations where AI outputs may conflict with physicians’ judgment. Relatedly, participants expressed a moral asymmetry in error tolerance: patients were more willing to accept mistakes made by physicians than errors attributed to AI. This was not necessarily because physicians were seen as more accurate but because they were perceived as accountable agents capable of explaining and justifying their decisions. This aligns with evidence showing that trust in AI remains closely tied to visible physician oversight and responsibility [39], and with broader research demonstrating that algorithmic decisions are evaluated differently from human decisions in terms of responsibility, blame, and moral judgment [50]. More broadly, participants described a shift from a traditionally dyadic model of decision-making between physicians and patients toward a more complex configuration involving additional actors, such as AI systems and their developers. However, the distribution of responsibility across these actors remained unclear and unresolved for both patients and physicians.

Another ethical consideration was the question of disclosure. In our data, patients did not uniformly demand to be informed about AI use. Instead, preferences varied. Some wanted disclosure because information supported a sense of control, helped them form their own view, and allowed them to decide whether they trusted AI involvement. Others saw AI as part of the physician’s internal process and considered detailed disclosure unnecessary as long as the physician remained responsible. This variation maps closely onto recent evidence showing that patients have specific but selective information needs regarding AI, particularly around provider oversight, performance, regulatory status, and its impact on care, with such information playing a key role in shaping trust and acceptance [38,51]. At the same time, our findings complicate a simplistic transparency narrative. Physicians expressed concerns that disclosing AI use or exposing unresolved discrepancies could confuse patients, amplify anxiety, and provoke overdiagnosis or overtreatment. While the literature supports the importance of transparency, our findings suggest that disclosure must be calibrated to context and purpose: not all information is equally useful, and more information does not automatically lead to better participation.

Building on these epistemic and ethical uncertainties, our findings suggest that AI does not simply redistribute tasks but may reconfigure the relational structure of clinical decision-making. Rather than displacing existing roles, it reinforces the centrality of physician judgment while introducing new forms of complexity in how patients engage with information and decisions. In this study, role-related uncertainty appeared to be associated less with the replacement of existing actors than with shifting boundaries between support, interpretation, and responsibility in AI-supported care.

Importantly, AI itself was not perceived as an autonomous actor but as a tool embedded within physician-led decision-making. Participants consistently described AI as providing additional input, rather than functioning as an independent decision-maker. This aligns with existing literature showing that clinicians tend to support AI for well-defined or repetitive tasks while remaining cautious in areas requiring contextual judgment and relational care [52,53]. More broadly, research on AI implementation highlights the importance of integrating such systems into existing clinical workflows rather than positioning them as substitutes for human expertise [43,54].

Relatedly, the role of physicians emerged not as diminished but as expanded. Participants described an increased need for interpretation, explanation, and oversight, with AI reinforcing rather than replacing clinical judgment. Our findings suggest that the perceived value of AI-generated information depended not only on the information itself but also on how it was interpreted and communicated within the clinical encounter. In participants’ accounts, physicians played a central role in explaining and contextualizing this information. This can be understood through the notion of relational expertise, which emphasizes that clinical knowledge is not merely technical but emerges through interaction, interpretation, and engagement with the patient [55]. At the same time, this expansion was not experienced as purely positive. Some physicians described situations in which AI introduced additional supervisory responsibilities without necessarily reducing their existing workload, particularly when AI-generated outputs still required human review while clinical responsibility remained with the physician. They were also expected to engage in the evaluation, development, and implementation of AI systems, extending their role beyond clinical care into technological and organizational domains, including involvement in broader institutional conditions such as infrastructure, resources, and implementation strategies [43,54,56].

Finally, the patient’s role appeared more ambivalent. While some participants expressed a desire for greater involvement, particularly through access to information, others preferred to rely on the physician and avoid additional complexity. Physicians likewise described patients as heterogeneous in their readiness and capacity to engage with complex information. This pattern is consistent with existing research in prostate cancer showing substantial variation in informational preferences and decision-making needs, as well as with studies indicating that patient involvement does not necessarily increase in the presence of AI-supported decision-making [22,38]. In participants’ accounts, greater access to information did not necessarily imply greater independence in the therapeutic relationship, as patients continued to position physicians as central to interpreting and contextualizing clinical information.

### Limitations

This study has several limitations. First, the patient survey relied on self-reported familiarity with AI rather than verified exposure. While this reflects participants’ own perceptions of their familiarity with AI, it does not allow us to determine their actual level of knowledge or prior exposure to AI-based technologies. Second, while the total number of approached patients could not be tracked, the survey was initiated 99 times, yielding 51 fully completed responses. Incomplete entries reflect technical errors, duplicate attempts, or participant withdrawal. Our recruitment method—including bedside visits and direct phone calls from trusted urologists—may also have introduced selection and social desirability biases. Patients who completed the survey may have had higher digital literacy or higher baseline institutional trust than those who declined or discontinued participation.

More broadly, the findings should be interpreted in terms of transferability rather than broad generalizability. Following Stalmeijer et al [57], transferability can be considered in terms of applicability, resonance, and theoretical relevance. Regarding applicability, this was a relatively small, context-specific study conducted among patients with localized prostate cancer and physicians involved in their care within a single Belgian university hospital. Patients generally had limited direct experience with AI and primarily discussed its anticipated use in a physician-supervised diagnostic context, whereas physicians approached AI from the perspective of their clinical experience and professional responsibilities, with several having already used AI-based tools. The findings therefore reflect these particular clinical, institutional, and experiential conditions, and may not be directly applicable to populations with different levels of AI exposure, other disease contexts, or settings in which AI is already routinely integrated into care. Transferability, however, does not require the same attitudes to be reproduced across settings. The epistemic, ethical, and role-related dimensions identified in this study may resonate with other clinical contexts characterized by diagnostic uncertainty, consequential health decisions, and continued physician responsibility. At a conceptual level, these dimensions may provide a framework for examining how uncertainty is negotiated when AI is introduced into clinical decision-making, and how existing relationships of trust shape this process. Transferability therefore concerns not whether patients and physicians elsewhere will express the same views, but whether these patterns help to understand AI integration in comparable clinical contexts. Future research should examine whether and how these patterns emerge in other clinical populations and health care settings, and how they evolve as patients and health care professionals gain direct experience with AI in routine clinical practice.

### Conclusions

Taken together, our findings suggest that the integration of AI in prostate cancer diagnostics should not be understood primarily in terms of acceptance, but in relation to how it reshapes the conditions under which trust is established and maintained within the patient-physician relationship. In this clinical context, the findings suggest that the integration of AI may reconfigure uncertainty across epistemic, ethical, and role-related dimensions, affecting how knowledge is interpreted, responsibility is negotiated, and professional and patient roles are understood.

A central conceptual implication of these findings concerns the role of trust in how participants understood and navigated these uncertainties. Patients continue to rely on physicians to interpret and contextualize AI-generated information, while physicians remain accountable for decisions involving systems they do not fully control. In this sense, the introduction of AI may make the relational foundations of care more salient, rather than displacing them. In comparable clinical contexts, the integration of AI-based diagnostic tools may therefore require careful attention to these uncertainties and to the relational conditions through which trust is maintained, including clear allocation of responsibility and appropriate support for health care professionals in their expanding roles related to AI supervision, interpretation, and communication. Training initiatives for health care professionals, alongside patient-centered communication approaches adapted to varying informational needs and preferences, also appear essential to ensure the acceptable and sustainable integration of AI into prostate cancer diagnostics.

## Supplementary material

10.2196/101751 Multimedia Appendix 1 Full questionnaire in French.

10.2196/101751 Multimedia Appendix 2 Patients focus group guide.

10.2196/101751 Multimedia Appendix 3 Physicians interview guide.

## Abbreviations

FLUTE: Federated Learning and Multi-Party Computation Techniques for Prostate Cancer
NASSS: nonadoption, abandonment, scale-up, spread, and sustainability
