# Supplementary material for: Patients’ and Physicians’ Perceptions of AI Integration in Prostate Cancer Diagnosis: Mixed Methods Study of Challenges to the Patient-Physician Relationship
Source: J Med Internet Res. 2026 Sep 30;28:e101751. doi: 10.2196/101751 (PMC13626195; doi:10.2196/101751)
Supplement: Supplementary file 1 [file jmir-v28-e101751-s001.pdf]

1. Questionnaire numéro 1 :

Vous êtes invité à participer à une enquête.
Avant que vous n'acceptiez de participer à cette étude, nous vous invitons à lire ce formulaire qui décrit les objectifs et les modalités pratiques.
Vous avez le droit de poser, à tout moment, des questions en rapport avec cette enquête auprès de la co-investigatrice de cette étude, Ekaterina Koshmanova au +32 4 323 33 50.

[Le formulaire d'information et de consentement est inséré ici]

Veuillez cocher les cases si vous êtes d'accord avec les énoncés. En cochant toutes les cases, vous consentez à l'étude et aurez alors accès au questionnaire.

- ☐ Je déclare que j'ai été informé sur la nature de l'étude, son but, sa durée, les effets secondaires éventuels et ce que l'on attend de moi. J'ai pris connaissance du document d'information et des annexes à ce document.
- ☐ J'ai eu l'occasion de poser toutes les questions qui me sont venues à l'esprit et j'ai obtenu une réponse satisfaisante à mes questions.
- ☐ J'ai compris que ma participation à cette étude est volontaire et que je suis libre de mettre fin à ma participation à cette étude sans qu'il soit nécessaire justifier ma décision et sans que cela ne n'entraine le moindre désavantage.
- ☐ Je consens de mon plein gré, sans avoir subi aucune pression physique ni psychologique.
- ☐ J'ai compris que des données me concernant seront récoltées pendant toute ma participation à cette étude et que l'investigateur et le promoteur se portent garant de la confidentialité de ces données, dans le respect de : la loi du 22 août 2002 relative aux droits du patient, la loi du 7 mai 2004 relative à l'expérimentation sur la personne humaine et les réglementations européennes et belges en vigueur (règlementation générale européenne sur la protection des données à caractère personnel [RGPD] du 25 mai 2018 et la loi belge du 30 juillet 2018 relative à la protection de la vie privée).
- ☐ Je consens au traitement de mes données personnelles selon les modalités décrites dans la rubrique traitant de garanties de confidentialité.
- ☐ J'ai été informé de l'existence d'une assurance
- ☐ Je sais que cette enquête a été soumise et approuvée par le Comité d'Ethique Hospitalo-Facultaire Universitaire de Liège - CEHFUL.
- ☐ J'ai reçu une copie de l'information au participant et du consentement éclairé. Pour obtenir cette copie, veuillez télécharger le document que vous venez de lire.

Si le participant coche toutes les cases, la suite s'ouvre. S'il ne coche pas toutes les cases, le questionnaire s'arrête.

Merci de cocher les cases si elles correspondent à votre situation personnelle :

- ☐ J'ai plus de 18 ans
- ☐ J'ai un cancer de la prostate non métastatique, c'est-à-dire que mon cancer est juste situé dans ma prostate et pas dans d'autres endroits (par exemple, les poumons, le foie, etc)
- ☐ Je ne suis pas en phase terminale du cancer
- ☐ Je n'ai pas de retard mental ou de démence

Si le participant coche toutes les cases, la suite s'ouvre. S'il ne coche pas toutes les cases, le questionnaire s'arrête.

Aujourd'hui, si un médecin pense qu'une personne pourrait avoir un **cancer de la prostate**, il lui demande de faire une **IRM**. L'IRM est une machine qui prend des **images de la prostate**. Ces images aident à décider s'il faut faire une **biopsie**. Une **biopsie** est un examen où les **médecins prélèvent un petit morceau de la prostate** pour l'examiner. Cela permet de savoir si :

- Il y a un cancer **signifiant** qui doit être **traité**.
- Il y a un cancer **insignifiant** qui **n'a pas besoin d'être traité,** mais qui **doit être**. Certains cancers évoluent si lentement qu'ils ne sont pas dangereux pour la personne atteinte.
- Il **n'y a pas** de cancer.

Aujourd'hui, il y a :

- Beaucoup de **personnes** qui font une **biopsie après une IRM** alors que **ce n'était pas nécessaire**.
- Beaucoup de **cancers insignifiants** qui ne **devraient pas être traités** mais **qui le sont** car on ne sait pas toujours si c'est un cancer qu'il faut traiter ou non.

Les **chercheurs** essaient donc de trouver des solutions pour mieux savoir :

- Si une **biopsie est nécessaire** après l'IRM **ou pas.**
- Si le **cancer** doit être **traité ou pas**.

Pour cela, les chercheurs essaient de construire des **machines** avec de **l'intelligence artificielle**. L'intelligence artificielle est une **technologie** qui aide les machines à faire des tâches que les humains font habituellement. Dans le domaine médical, elle peut **aider les médecins à être plus efficaces**.

L'**intelligence artificielle pourra aidera les médecins** à regarder les images de la prostate prises par IRM. Cela les **aidera** à

- **Décider s'il faut faire une biopsie après l'IRM ou pas**. Cela permettra de réduire le nombre de biopsies inutiles. **Pourquoi est-ce important ?** Parce qu'une biopsie peut être désagréable et comporter des risques, comme des infections ou des saignements.
- **Déterminer si le cancer est insignifiant ou s'il qui doit être traité.** Cela **évitera** que des personnes soient **traitées inutilement** et subissent les **effets secondaires** du traitement.

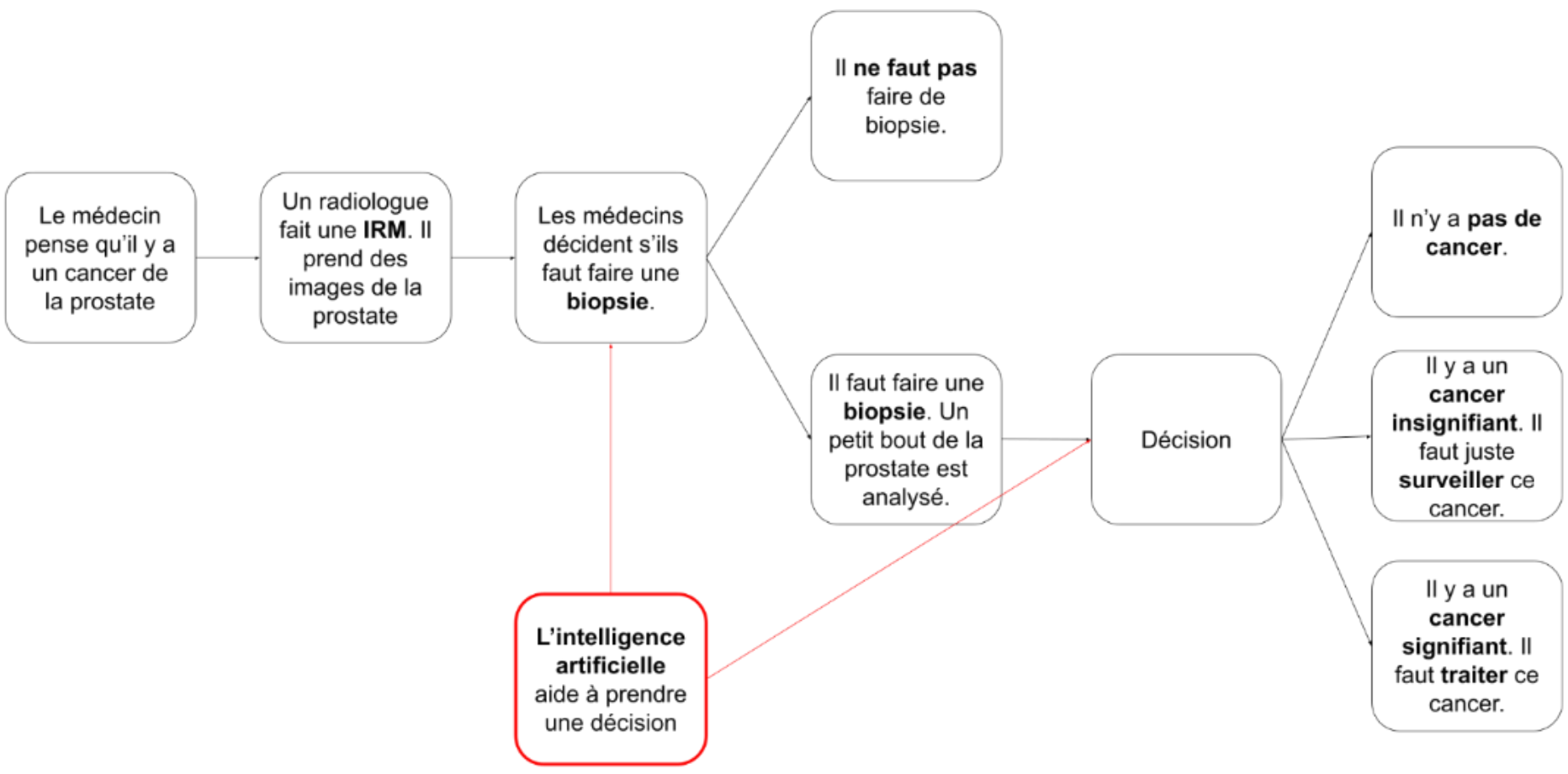

Dans notre étude, nous **ne vous demandons pas de tester** les machines d'intelligence artificielle. Nous voulons plutôt **connaître votre opinion** sur ces machines d'intelligence artificielle utilisées pour aider les prestataires de soins de santé à diagnostiquer le cancer de la prostate.

1. La règlementation vous permet de retirer votre consentement à l'étude à tout moment. Pour cela, veuillez créer un code avec lequel nous pourrons identifier vos réponses si vous décidez de ne plus participer à l'étude.
   Ce code doit être composé comme cela : la première lettre de votre nom de famille + la dernière lettre de votre nom de famille + la première lettre de votre prénom + les 2 derniers chiffres de votre année de naissance.

   Par exemple, si je suis **D**UPON**T** **J**ean, né le 02.09.19**60**, mon code est : **dtj60**

   Veuillez écrire votre propre code :

…….

2. Suite aux explications que je viens de lire, je comprends comment l'intelligence artificielle aidera à améliorer le diagnostic du cancer de la prostate :

	1	2	3	4	5	
Pas du tout d'accord	○	○	○	○	○	Tout à fait d'accord

3. Je pense que les outils basés sur l'intelligence artificielle peuvent améliorer le diagnostic du cancer de la prostate :

	1	2	3	4	5	6	7	
Pas du tout d'accord	○	○	○	○	○	○	○	Tout à fait d'accord

4. Je pense que les outils basés sur l'intelligence artificielle peuvent faire progresser le processus de diagnostic du cancer de la prostate :

	1	2	3	4	5	6	7	
Pas du tout d'accord	○	○	○	○	○	○	○	Tout à fait d'accord

5. Je pense que les outils basés sur l'intelligence artificielle peuvent proposer un diagnostic précis du cancer de la prostate :

	1	2	3	4	5	6	7	
Pas du tout d'accord	○	○	○	○	○	○	○	Tout à fait d'accord

6. Je pense que les outils basés sur l'intelligence artificielle peuvent réduire les coûts de diagnostic du cancer de la prostate :

	1	2	3	4	5	6	7	
Très faible	○	○	○	○	○	○	○	Très élevé

7. Le risque d'utiliser des outils basés sur l'intelligence artificielle à des fins de diagnostic du cancer de la prostate est :

	1	2	3	4	5	6	7	
Très faible	○	○	○	○	○	○	○	Très élevé

8. Le degré d'incertitude associé à l'utilisation d'outils cliniques basés sur l'intelligence artificielle à des fins de diagnostic du cancer de la prostate est :

	1	2	3	4	5	6	7	
Très faible	○	○	○	○	○	○	○	Très élevé

9. La perte potentielle associée à l'utilisation d'outils basés sur l'intelligence artificielle à des fins de diagnostic du cancer de la prostate est :

	1	2	3	4	5	6	7	
Très faible	○	○	○	○	○	○	○	Très élevé

10. La probabilité de problèmes inattendus liés à l'utilisation d'outils basés sur l'intelligence artificielle à des fins de diagnostic du cancer de la prostate est :

	1	2	3	4	5	6	7	
Très faible	○	○	○	○	○	○	○	Très élevé

11. Globalement, la probabilité de conséquences indésirables associées à l'utilisation d'outils basés sur l'intelligence artificielle à des fins de diagnostic du cancer de la prostate est :

	1	2	3	4	5	6	7	
Très faible	○	○	○	○	○	○	○	Très élevé

12. J'aurais aimé utiliser des outils basés sur l'intelligence artificielle pour gérer mon diagnostic de cancer de la prostate :

- o Oui
- o Incertain car (veuillez indiquer sur une échelle de 0 à 10 quelle est l'importance des craintes suivantes) :
- o Non car (veuillez indiquer sur une échelle de 0 à 10 quelle est l'importance des craintes suivantes) :

13. Cette question sera ouverte uniquement si la réponse à la question 12 est « Incertain car (veuillez indiquer sur une échelle de 0 à 10 quelle est l'importance des craintes suivantes) » ou « Non car (veuillez indiquer sur une échelle de 0 à 10 quelle est l'importance des craintes suivantes) »
    - Je crains que l'intelligence artificielle donne un diagnostic du cancer de la prostate inexacte.
    - Je crains que l'intelligence artificielle puissent diminuer les aspects humains des relations.
    - Je crains que l'intelligence artificielle me mette dans une position plus passive pour prendre des décisions médicales concernant le diagnostic du cancer de la prostate.
    - Je crains que la confidentialité de mes informations de santé ne soit pas respectée lors de l'utilisation de l'intelligence artificielle.
    - Je ne fais pas confiance aux machines qui utilisent l'intelligence artificielle pour aider au diagnostic du cancer de la prostate.
    - Je n'ai pas confiance dans le fait que les machines qui utilisent l'intelligence artificielle puissent s'adapter à des situations médicales spécifiques et imprévues.
    - Je crains que la sécurité et l'efficacité des machines qui utilisent l'intelligence artificielle ne soient pas clairement réglementées.

- Je suis inquiet de savoir qui devient responsable si l'intelligence artificielle donnent de mauvaises recommandations pour le diagnostic du cancer de la prostate.
- Je crains que l'intelligence artificielle entraîne des coûts de soins de santé plus élevés.
- Si la réponse à la question 12 est « Incertain car (veuillez indiquer sur une échelle de 0 à 10 quelle est l'importance des craintes suivantes) », cette proposition s'ouvre : Je crains ou je suis préoccupé par autre chose que les propositions précédentes : (veuillez indiquer quoi):

  ……

  Si la réponse à la question 12 est « Non car (veuillez indiquer sur une échelle de 0 à 10 quelle est l'importance des craintes suivantes) », cette proposition s'ouvre : J'ai une ou plusieurs autre(s) raison(s) de refus que les propositions précédentes (Veuillez indiquer quoi) :

  ……

14. Les personnes qui influencent mon comportement pensent que je devrais utiliser des outils basés sur l'intelligence artificielle pour le diagnostic du cancer de la prostate :

	1	2	3	4	5	6	7	
Pas du tout d'accord	○	○	○	○	○	○	○	Tout à fait d'accord

15. Les personnes qui sont importantes pour moi pensent que je devrais utiliser des outils basés sur l'intelligence artificielle pour le diagnostic du cancer de la prostate :

	1	2	3	4	5	6	7	
Pas du tout d'accord	○	○	○	○	○	○	○	Tout à fait d'accord

16. En général, les médecins soutiendront l'utilisation des outils basés sur l'intelligence artificielle pour le diagnostic du cancer de la prostate :

	1	2	3	4	5	6	7	
Pas du tout d'accord	○	○	○	○	○	○	○	Tout à fait d'accord

17. Pour se développer, l'intelligence artificielle a besoin d'accéder à une grande quantité de données de santé. Pour cela, les personnes doivent accepter de fournir leurs données de santé. Lorsque quelqu'un accepte de fournir ses données de santé, celles-ci sont anonymes. Imaginez qu'on vous demande si vous accepteriez de rendre vos données de santé disponibles pour le développement de l'intelligence artificielle dans le secteur médical. Accepteriez-vous ?

- o Oui, j'accepterais sans restriction.
- o Oui, j'accepterais mais uniquement sous certaines conditions.
- o Non, je n'accepterais pas.

18. Cette question sera ouverte uniquement si la réponse à la question 17 est « Oui, j'accepterais mais uniquement sous certaines conditions :
    Veuillez indiquer dans quelles conditions vous accepteriez de rendre vos données de santé disponibles pour le développement de l'intelligence artificielle dans le secteur médical :

……….

19. Cette question sera ouverte uniquement si la réponse à la question 17 est Non, je n'accepterais pas :
    Veuillez indiquer pourquoi vous n'accepteriez de pas de rendre vos données de santé disponibles pour le développement de l'intelligence artificielle dans le secteur médical :

……

20. Selon vous, quel est le niveau de risque associé au fait de rendre vos données de santé disponibles pour le développement de l'intelligence artificielle dans le secteur médical ?

	1	2	3	4	5	
Très faible	○	○	○	○	○	Très élevé

21. Quels sont, selon vous, les risques liés au fait de rendre vos données de santé disponibles pour le développement de l'intelligence artificielle dans le domaine médical ?

……

22. Selon vous, quel est le niveau de bénéfice associé au fait de rendre vos données de santé disponibles pour le développement de l'intelligence artificielle dans le secteur médical ?

	1	2	3	4	5	
Très faible	○	○	○	○	○	Très élevé

23. Quels sont, selon vous, les bénéfices liés au fait de rendre vos données de santé disponibles pour le développement de l'intelligence artificielle dans le domaine médical ?

……

Questions générales :

24. Quel âge avez-vous ?

……

25. Dans quel pays vivez-vous ?
- o Belgique
- o Italie
- o Espagne
- o Autre

26. Laquelle des descriptions suivantes se rapproche le plus de ce que vous ressentez concernant le revenu de votre ménage actuellement ?
- o Je vis confortablement avec le revenu actuel de mon ménage
- o Je m'en sors avec le revenu actuel de mon ménage
- o Je trouve difficile de s'en sortir avec le revenu actuel de mon ménage
- o Je trouve très difficile de s'en sortir avec le revenu actuel de mon ménage
- o Je ne sais pas

27. Quel est votre statut professionnel ?
- o Employé à plein temps
- o Employé à temps partiel
- o Chômeur
- o Retraité
- o Étudiant

28. Quel est le plus haut niveau d'éducation que vous avez obtenu ?

- Aucun
- Enseignement primaire
- Enseignement secondaire inférieur
- Enseignement secondaire supérieur
- Enseignement supérieur non-universitaire
- Enseignement universitaire
- Doctorat

29. Comment évalueriez-vous votre état de santé ?

- Très bon
- Bon
- Passable
- Médiocre
- Très médiocre

30. Quelle est votre situation ?

- Je ne suis pas de traitement pour le cancer de la prostate, mais mon cancer est régulièrement surveillé, par exemple, par une IRM, une biopsie ou une analyse de sang.
- Je suis un traitement pour le cancer de la prostate, par exemple par radiothérapie ou par chirurgie.
- Je ne sais pas.

31. Depuis combien de temps avez-vous été diagnostiqué avec un cancer de la prostate ? (Veuillez indiquer le mois et l'année de votre diagnostique. Si vous ne vous rappelez pas du mois, veuillez indiquer "Janvier" puis l'année de votre diagnostique) :

………

32. Au cours des 12 derniers mois, en excluant les visites aux urgences, combien de fois avez-vous consulté un médecin, une infirmière ou un autre professionnel de santé ?

………

33. Avez-vous déjà utilisé des services de santé utilisant l'intelligence artificielle ? (Comme l'intelligence artificielle intégrée dans des outils médicaux intelligents)

- Oui
- Non
- Je ne sais pas

34. Comment évalueriez-vous vos connaissances techniques sur l'intelligence artificielle ?

- Terribles
- Pauvres
- Moyennes
- Bonnes
- Excellentes

35. Quelle est votre familiarité avec le sujet de l'intelligence artificielle ?

- Complètement non familier
- Non familier
- Neutre

- o Familier
- o Très familier

36. Comment évalueriez-vous vos expériences de soins antérieures ?

- o Très bonnes
- o Bonnes
- o Passables
- o Médiocres
- o Très médiocres

37. Les institutions de santé se préoccupent uniquement de réduire les coûts médicaux, et non de ce dont j'ai besoin pour ma santé.

	1	2	3	4	5	
Pas du tout d'accord	○	○	○	○	○	Tout à fait d'accord

38. Les institutions de santé offrent la meilleure qualité de soins médicaux.

	1	2	3	4	5	
Pas du tout d'accord	○	○	○	○	○	Tout à fait d'accord

39. Lors du traitement de mes problèmes médicaux, les institutions de santé mettent mes besoins médicaux au-dessus de toutes les autres considérations, y compris les coûts.

	1	2	3	4	5	
Pas du tout d'accord	○	○	○	○	○	Tout à fait d'accord

Littéracie :

40. Avez-vous besoin d'aide pour lire les documents de l'hôpital ?

- o Jamais
- o Rarement
- o Parfois
- o Souvent
- o Toujours

41. Je sais comment trouver des ressources de santé utiles sur internet

	1	2	3	4	5	
Pas du tout d'accord	○	○	○	○	○	Tout à fait d'accord

42. Je sais comment utiliser internet pour répondre à mes questions de santé

	1	2	3	4	5	
Pas du tout d'accord	○	○	○	○	○	Tout à fait d'accord

43. Je sais quelles sont les ressources de santé disponibles sur internet

	1	2	3	4	5	
Pas du tout d'accord	○	○	○	○	○	Tout à fait d'accord

44. Je sais où trouver des ressources de santé utiles sur Internet.

	1	2	3	4	5	
Pas du tout d'accord	○	○	○	○	○	Tout à fait d'accord

45. Je sais comment utiliser les informations de santé que je trouve sur Internet pour m'aider.

	1	2	3	4	5	
Pas du tout d'accord	○	○	○	○	○	Tout à fait d'accord

46. J'ai les compétences nécessaires pour évaluer les ressources de santé que je trouve sur Internet.

	1	2	3	4	5	
Pas du tout d'accord	○	○	○	○	○	Tout à fait d'accord

47. Je peux distinguer les ressources de santé de haute qualité de celles de basse qualité sur Internet.

	1	2	3	4	5	
Pas du tout d'accord	○	○	○	○	○	Tout à fait d'accord

48. Je me sens confiant dans l'utilisation des informations provenant d'Internet pour prendre des décisions de santé.

	1	2	3	4	5	
Pas du tout d'accord	○	○	○	○	○	Tout à fait d'accord

49. Pouvons-nous vous contacter pour vous inviter à participer à la deuxième phase de cette étude ?
    Cette deuxième phase consiste en un entretien de groupe sur le même sujet que le présent questionnaire. Si vous souhaitez être contacté, nous pourrons vous fournir toutes les informations nécessaires pour poursuivre le projet. Vous pourrez alors décider si vous souhaitez ou non participer au projet.
    Si vous acceptez d'être recontacté, vous le serez dans le courant du mois d'avril 2025.
    Souhaitez-vous être contacté ?
    o Oui.
    o Non.
50. Cette question ne sera ouverte que si la réponse à la question 50 est « oui » : Pour être contacté, veuillez cliquer sur ce lien : [le lien du deuxième questionnaire sera inséré]

2. Questionnaire numéro 2 :

Vous venez d'accepter d'être contacté pour participer à la deuxième phase de l'étude. Pour être contacté, veuillez fournir :

1. Votre prénom et nom :

……….

2. Votre numéro de téléphone :

……….

3. Le pays dans lequel vous vivez :

- o Belgique
- o Italie
- o Espagne
- o Autre

4. Votre code postal :

………..
