# Supplementary material for: Patients’ and Physicians’ Perceptions of AI Integration in Prostate Cancer Diagnosis: Mixed Methods Study of Challenges to the Patient-Physician Relationship
Source: J Med Internet Res. 2026 Sep 30;28:e101751. doi: 10.2196/101751 (PMC13626195; doi:10.2196/101751)
Supplement: Supplementary file 2 [file jmir-v28-e101751-s002.docx]

**Interview Guide — Patients**

**Introduction**

- Greetings and thanks for your participation.
- Introduction of the interviewer and the study framework (European project FLUTE).
- Objective: to understand your experience of the perception of AI in the diagnosis and treatment of prostate cancer.

In the first phase of our research, we collected patients’ perceptions and expectations regarding prostate cancer through an online questionnaire. Based on these initial results, this second phase aims to explore your point of view in more detail and to better understand how you perceive the integration of AI in your care pathway and your relationship with your doctor.

- Confidentiality and consent (recording of the interview).
- Duration: approximately 1.5 hours, series of open-ended questions, focus on your personal experience.
- Preliminary question: “Before we start, do you have any questions?”

**Theme 1: Your care pathway**

**General question:** (perhaps start by asking how their care pathway is going)
Can you tell me about your interactions with healthcare professionals from the first symptoms to diagnosis and treatment? What has been most important to you in these interactions?

**Probes:**

- Can you describe a moment that particularly marked you, either positively or negatively?
- Did you feel listened to and understood? Why or why not?
- Were the treatment options presented and discussed with you? Did you feel that your opinion was considered in the decision? (so we can discuss shared decision-making afterward)

**Theme 2: AI in your pathway — benefits and risks**

**Explanatory introduction:**
As a reminder, our study focuses on how artificial intelligence can be used to improve the diagnosis of patients with prostate cancer, while understanding how patients perceive the use of their health data in this context.

AI in prostate cancer – a brief explanation, what does the FLUTE project do?

- Protect your health data while enabling research.
  With the FLUTE platform, researchers can train AI models without moving your data from the hospital. The data remain protected on-site, preventing access by private companies. This allows care to be improved while respecting your privacy.
- Better predict aggressive prostate cancers.
  We are developing a decision-support tool that combines multiple sources of information (blood tests, medical history, imaging, etc.) to determine whether a biopsy is necessary.
  Today, this tool could help avoid about 1 in 4 biopsies. We are still working to improve it, notably by adding markers from imaging.

**Introduction to the questions:**
In the first part of our study, participants explained what they saw as the advantages and risks of artificial intelligence (AI) in medicine. Many believe that AI could help doctors make diagnoses faster and more accurately. Most are therefore supportive of its use.

However, several participants also expressed concerns. They worry, for example, that the use of AI could reduce the human relationship with their doctor, or that it may not be clear who would be responsible in case of an error: the machine or the doctor? Other common questions include: will their data remain confidential? And who will actually make the final decision: the AI or the doctor?

N = 42, Age = 67 ± 7.9

I would like to use artificial intelligence-based tools to manage my prostate cancer diagnosis.

|  |  |
| --- | --- |
| Yes | 20 |
| Uncertain | 20 |
| No | 1 |

N = 21, Age = 65 ± 8.2

**Main Questions:**

1. Have you ever heard about the use of AI in medical diagnosis?
   **Probes:**

- If yes, can you tell me what you know about it?
- If no, what would be your first impression or concern?

2. What advantages do you think AI could bring to your medical pathway?
   **Probes:**

- Could it improve diagnostic accuracy?

3. What risks or concerns seem most important to you regarding AI?
   **Probes:**

- Are you afraid of errors in diagnosis or treatment?
- Do you worry about the confidentiality of your health data?
- Could the use of AI complicate your care or your understanding?

4. Would you like to be informed if your doctor uses AI-based tools?
   **Probes:**

- If yes, how? Do you prefer a simple explanation, a detailed explanation, or a written document?
- What information is most important for you?

5. In your opinion, who should be responsible in case of an error in your diagnosis or treatment after the use of AI?
   **Probes:**

- Do you think the responsibility should lie with the doctor, the AI developer, or both?
- Would this change your confidence in the treatment?

**Theme 3: Impact on your relationship with the doctor**

**Main Questions:**

1. How do you think AI could influence communication and trust with your doctor?
   **Probes:**

- Could AI strengthen or weaken your relationship with the doctor?
- Are there situations where AI could be beneficial for discussion with the doctor?

2. Are you concerned about a loss of the human connection with your doctor? Why?
   **Probes:**

- Does this concern change depending on the type of medical decision?
- Do you have examples where the human relationship was particularly important?

3. How could AI support the doctor-patient relationship?
   **Probes:**

- Could AI facilitate discussion about treatment options?
- Could it help better understand your results and medical decisions?

4. How would you like AI to be integrated into shared medical decisions?
   **Probes:**

- Do you want to be consulted at every step or only for certain decisions?
- What information should be available to you in order to participate fully?

**Theme 4: Conditions for AI to be acceptable to you**

**Main Questions:**

1. What conditions must be met for the use of AI to be safe and acceptable in your care?
   **Probes:**

- Transparency about how AI works?
- Training and competence of doctors using AI?
- Guarantees of data security and confidentiality?

2. What information or resources would help you better understand and accept AI?
   **Probes:**

- Simple explanations, brochures, videos?
- Direct discussion with the doctor or a specialized healthcare professional?

**Closing:**

- Collection of participant characteristics: age, country, current treatment, familiarity with AI, previous experience with healthcare, etc.
- Thanks: “Thank you very much for your participation. Do not hesitate to contact us for any questions or comments.”
