# Supplementary material for: Patients’ and Physicians’ Perceptions of AI Integration in Prostate Cancer Diagnosis: Mixed Methods Study of Challenges to the Patient-Physician Relationship
Source: J Med Internet Res. 2026 Sep 30;28:e101751. doi: 10.2196/101751 (PMC13626195; doi:10.2196/101751)
Supplement: Supplementary file 3 [file jmir-v28-e101751-s003.docx]

**Interview Guide — Healthcare Professionals**

**Introduction**

- Greetings and thanks for your participation.
- Introduction of the interviewer, the research framework (European FLUTE project), and the objectives of the study.
- CHU Liège is part of the EU-funded FLUTE project, which aims to develop an AI-supported platform enabling physicians to analyze suspected prostate cancer cases without prescribing invasive tests such as prostate biopsy.
- Objective: to better understand the acceptability of AI, and the conditions surrounding it, among people with prostate cancer and healthcare professionals in the context of prostate cancer diagnosis and AI as a support tool for healthcare providers.
- Confidentiality and consent (recording of the interview).
- Duration: approximately 1 hour, interview consisting of a series of open-ended questions, focus on personal experience and perceptions.
- Preliminary question: “Before we begin the interview, do you still have any questions?”

**Theme 1: Clinical Pathway**

General question: At what stage of the patient’s diagnostic pathway do you intervene? What elements are the most sensitive or important in communication with your patients?

Probes:

- Can you describe the interactions you have with patients during the pre-diagnostic period?
- What is most important to you in these interactions?
- Do you think patients feel listened to and understood during the first consultations? Why or why not?
- If you could improve certain elements at this stage of the care pathway, what would they be and why?

**Theme 2: Integration of AI — Benefits and Risks**

Explanatory introduction:

As a reminder, our study focuses on how artificial intelligence can be used to improve the diagnosis and treatment of prostate cancer, while understanding how healthcare professionals perceive the integration of AI into their clinical practice and the doctor–patient relationship.

AI in prostate cancer – brief explanation:

AI could help physicians refine MRI interpretation, determine whether a biopsy is necessary, and distinguish clinically significant cancers from insignificant ones. This could reduce unnecessary biopsies and treatments while improving diagnostic precision. However, AI also presents several risks, including data confidentiality issues, diagnostic errors or algorithmic bias, and the complexity of integrating these systems into clinical workflows.

Introduction to the questions: in the first part of our study, patients identified potential benefits of AI, especially improved diagnostic accuracy, but also concerns regarding responsibility in case of errors, trust in AI systems, confidentiality of health data, and the possible loss of the human relationship with physicians.

N = 42, Age = 67 ± 7.9

I would like to use artificial intelligence-based tools to manage my prostate cancer diagnosis.

|  |  |
| --- | --- |
| Yes | 20 |
| Uncertain | 20 |
| No | 1 |

N = 21, Age = 65 ± 8.2

Main Questions:

1. Have you already used artificial intelligence in your clinical practice? If yes, how and in which situations? If no, why not?

2. What is your current experience or practice with AI in prostate cancer diagnosis?

3. Do you inform your patients when you use AI-based tools? If yes, how?

4. What concerns or fears do you personally have regarding the use of AI?

5. In your opinion, who should be responsible in the event of an AI-related error?

Ease of use:

· To what extent would it be easy for you to integrate AI into your current practice?

· What difficulties do you foresee in the use of AI?

**Theme 3: Impact on the Doctor–Patient Relationship**

Main Questions:

1. In your opinion, how could AI influence communication, trust, and the physician’s role with patients?

Probes:

- Could AI strengthen or weaken the doctor–patient relationship?
- In what ways could AI support the human connection between patient and physician?

2. Patients express concerns that AI could lead to a loss of the human relationship in care. In your opinion, are these concerns justified? Why?

3. What role could AI play in shared medical decision-making?

**Theme 4: Conditions and Resources for the Integration of AI**

Main Questions:

1. In an ideal world, what conditions would be essential for the successful integration of AI?

Probes:

- Transparency regarding how AI works?
- Training and competence of healthcare professionals using AI?
- Guarantees of data security and confidentiality?

2. What current resources could facilitate its integration?

3. What resources are still lacking to achieve this?

**Closing**

- Collection of participant characteristics: sex, years of experience in prostate cancer care, country, clinical specialization, familiarity with AI, previous use of AI in healthcare, etc.
- Thanks: “We have now reached the end of this interview. Thank you very much for your participation. Please do not hesitate to contact us if you have any questions or comments.”
